# Insights into the pathogenesis of gestational and hepatic diseases: the impact of ferroptosis

**DOI:** 10.3389/fcell.2024.1482838

**Published:** 2024-11-12

**Authors:** Yilan You, Zhiwen Qian, Ying Jiang, Lingyan Chen, Danping Wu, Lu Liu, Feng Zhang, Xin Ning, Yan Zhang, Jianping Xiao

**Affiliations:** ^1^ Departments of Obstetrics and Gynecology, Wuxi Maternal and Child Healthcare Hospital, Wuxi Medical Center, Nanjing Medical University, Wuxi, China; ^2^ Departments of Obstetrics and Gynecology, Wuxi Maternity and Child Healthcare Hospital, Women’s Hospital of Jiangnan University, Jiangnan University, Wuxi, China

**Keywords:** ferroptosis, pregnancy, placenta, liver diseases, pathogenesis

## Abstract

Ferroptosis, a distinct form of non-apoptotic cell death characterized by iron dependency and lipid peroxidation, is increasingly linked to various pathological conditions in pregnancy and liver diseases. It plays a critical role throughout pregnancy, influencing processes such as embryogenesis, implantation, and the maintenance of gestation. A growing body of evidence indicates that disruptions in these processes can precipitate pregnancy-related disorders, including pre-eclampsia (PE), gestational diabetes mellitus (GDM), and intrahepatic cholestasis of pregnancy (ICP). Notably, while ICP is primarily associated with elevated maternal serum bile acid levels, its precise etiology remains elusive. Oxidative stress induced by bile acid accumulation is believed to be a significant factor in ICP pathogenesis. Similarly, the liver’s susceptibility to oxidative damage underscores the importance of lipid metabolism dysregulation and impaired iron homeostasis in the progression of liver diseases such as alcoholic liver disease (ALD), non-alcoholic fatty liver disease (NAFLD), cholestatic liver injury, autoimmune hepatitis (AIH), acute liver injury, viral hepatitis, liver fibrosis, and hepatocellular carcinoma (HCC). This review discusses the shared signaling mechanisms of ferroptosis in gestational and hepatic diseases, and explores recent advances in understanding the mechanisms of ferroptosis and its potential role in the pathogenesis of gestational and hepatic disorders, with the aim of identifying viable therapeutic targets.

## 1 Introduction

Programmed cell death (PCD) is a genetically regulated process essential for maintaining body homeostasis and is involved in various pathological conditions. The PCD pathways encompass apoptosis, autophagy, pyroptosis, ferroptosis, among others. Ferroptosis, a recently identified non-apoptotic form of PCD characterized by iron-dependent oxidative damage, has garnered significant attention due to its involvement in a wide range of pathological conditions (Dixon et al., 2012). Although it often interacts with other PCD pathways to induce cell destruction, ferroptosis is distinct in its morphological, biochemical, and genetic characteristics (Zhang et al., 2023) (Table 1).

**TABLE 1:** Characteristics of different PCDs.

	Inducement	Morphological feature	Biochemical feature	Regulator
Apoptosis	Modification of the intracellular environment	Smaller volume of the cell, intact cell membrane, formation of the apoptotic vesicle, condensed and marginalized chromatin	DNA fragmentation, ectopic phosphatidylserine on the cell membrane surface, elevated lysosomal and endolysosomal enzyme levels, and caspase activation	Bcl-2 family, Fas, TNFR1, Caspase, and P53
Ferroptosis	Iron accumulation and lipid peroxidation	Reduced cell volume, intact cell membrane, apoptotic vesicle formation, and condensed, marginalized chromatin	Intracellular glutathione (GSH) depletion, reduced activity of glutathione peroxidase 4 (GPX4), and an overwhelming, iron-dependent buildup of lethal lipid ROS	System Xc^−^, erastin, GPX4, PUFAs, IREB2, SLC7A11, P53, FSP1, NOX, NCOA4
Autophagy	Nutrient deprivation, hypoxia, pathogen infection, and endoplasmic reticulum stress	Initiation of phagophore, formation of autophagosomes and autolysosomes, double-membraned autophagosomes, encapsulating cytoplasmic components	Production of autophagy-related gene (ATG) family proteins and the conversion of microtubule-associated protein light chain 3 (LC3) from type I to type III	ULK1 complex, PI3KC3 complex, ALG12-ATG5-ATG16 complex, Rabs, and HOPS complex
Pyroptosis	Infection by pathogen	Cells swell forming protrusions, while the cell membrane develops pores	Formation of inflammatory vesicles, activation of caspase and gasdermin, and release of inflammatory factors	Caspase, GSDMs, NLPP1, and NLRP3
Necroptosis	Physical factors such as high temperatures and radiation, chemical factors like strong acids, alkalis, and toxic substances, as well as biological factors including bacteria and viruses	Plasma membrane rupture, organelle swelling, and moderate chromatin condensation	Randomly degraded DNA fragments independent of ATP	RIP1/3 and MLKL

Ferroptosis impacts cellular susceptibility through its involvement in key metabolic pathways, including (seleno)thiol metabolism, fatty acid metabolism, iron metabolism, the mevalonate pathway, and mitochondrial respiration (Zheng and Conrad, 2020). The deficiency of membrane lipid repair enzymes, particularly glutathione peroxidase 4 (GPX4), leads to the accumulation of reactive oxygen species (ROS) in membrane lipids. Modulating ferroptosis is anticipated to impede the progression of diseases related to lipid metabolism. Among these, gestational and hepatic diseases stand out as critical areas where ferroptosis plays a pivotal role. Ferroptosis has been implicated in the pathogenesis of both gestational and hepatic diseases through mechanisms involving iron dysregulation, oxidative stress, and lipid peroxidation. The relaation between gestational and hepatic diseases can be exemplified by conditions such as intrahepatic cholestasis of pregnancy (ICP), which directly links pregnancy with liver dysfunction. ICP is a gestational liver disorder characterized by impaired bile flow, leading to elevated bile acid levels in the maternal circulation. This condition not only manifests in hepatic symptoms, such as pruritus and jaundice, but also poses significant risks to fetal health, including preterm birth, fetal distress, and stillbirth. The occurrence of ICP underscores the critical interplay between pregnancy and liver function, illustrating how gestational diseases can have direct hepatic implications. This connection is particularly important in the context of ferroptosis, as both gestational liver diseases and traditional hepatic disorders may share overlapping pathological mechanisms, including dysregulation of lipid peroxidation, iron metabolism, and oxidative stress. Recognizing these shared pathways highlights the need for an integrated approach to understanding ferroptosis in both pregnancy-related and liver-specific contexts. Understanding the shared signaling mechanisms of ferroptosis in these conditions is crucial for developing targeted therapies, as modulating ferroptosis may hold therapeutic potential across both disease spectra.

Given the growing body of evidence linking ferroptosis to the progression of liver and pregnancy-related diseases, this review aims to explore the common pathways and potential therapeutic targets. By examining the interplay between ferroptosis and these diseases, we hope to shed light on novel strategies for treatment, making this a timely and highly relevant topic in the field of biomedical research.

## 2 Ferroptosis

### 2.1 Discovery of ferroptosis

In 2003, Dolma et al. utilized synthetic lethal high-throughput screening to discover compounds with genotype-selective cytotoxicity, specifically targeting tumor cells harboring certain oncoproteins or lacking specific tumor suppressors. This approach led to the identification of erastin, a small molecule, lethal to cells expressing RAS and ST, with erastin-induced cell death being non-apoptotic (Dolma et al., 2003). Following this, in 2007, Yagoda et al. revealed that erastin exerts its antitumor effects *via* mitochondrial voltage-dependent anion channels (VDACs), with VDAC2 and VDAC3 being essential, though not solely sufficient, for erastin-induced cell death (Yagoda et al., 2007). In 2009, Yang identified two compounds, RSL3 and RSL5, which selectively induce cell death in obcogenic RAS-expressing cells. This cell death mechanism was notably distinct from apoptosis, as it was not regulated by caspase inhibitors but could be inhibited by iron chelators and antioxidants (Yang and Stockwell, 2008). In 2012, Dixon et al. coined the term “ferroptosis” to describe this erastin-triggered form of cell death, characterized primarily by mitochondrial volume reduction, mitochondrial membrane ruffling, outer membrane rupture, and loss of cristae (Dixon et al., 2012). Erastin inhibits the cystine/glutamate antiporter system Xc^−^, resulting in compromised cellular antioxidant defenses, toxic ROS accumulation, and lipid-peroxidized membrane disruption, all leading to oxidative cell death, dependent on intracellular iron (Dixon et al., 2012). Further research found that erastin-induced ferroptosis also elevates levels of lysosome-associated membrane protein 2a, thereby promoting chaperone-mediated autophagy and subsequent GPX4 degradation (Wu et al., 2019). In 2014, Yang et al. demonstrated that glutathione (GSH) depletion is pivotal in erastin-induced ferroptosis, as it inactivates glutathione-dependent peroxidases (GPXs), key enzymes for cellular membrane lipid repair, whose deficiency results in ROS accumulation in membrane lipids (Yang et al., 2014). Besides RSL3, several chloroacetamide-containing GPX4 inhibitors were also identified, including DPI7/ML162, DPI6, DPI8, DPI9, DPI12, DPI13, DPI15 and DPI19 (Yang et al., 2014).

This reflection on the chronological development of ferroptosis research underscores the evolution of our understanding of this distinct form of cell death and its potential implications for cancer therapy. The identification of specific molecules and mechanisms has opened new avenues for targeted therapies, emphasizing the importance of continued research in this field.

### 2.2 Mechanism of ferroptosis

#### 2.2.1 Iron metabolism

**FIGURE 1:**
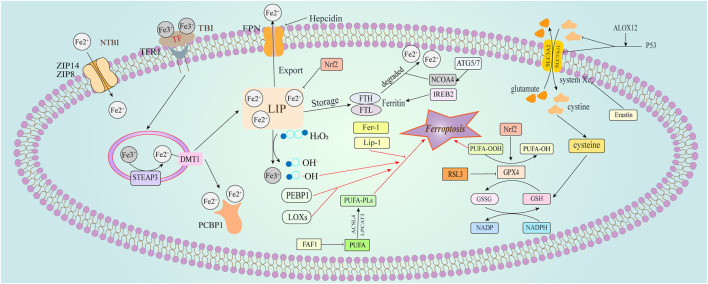
Physiological cellular iron transport and regulation of ferroptosis. This figure illustrates the pathways involved in cellular iron transport and the regulatory mechanisms of ferroptosis. Iron is primarily transported as transferrin-bound iron (TBI). Plasma-derived transferrin (TF) binds to transferrin receptor 1 (TFR1) on the cell membrane, leading to receptor-mediated endocytosis of the TF-TFR1 complex. Within the acidified endosome, iron is released from TF after reduction by six-transmembrane epithelial antigen of the prostate 3 (Steap3), then transported to the cytoplasm *via* divalent metal transporter 1 (DMT1). Intracellular iron is either sequestered by ferritin or mobilized where needed. Excess iron, forming non-transferrin-bound iron (NTBI) in overloaded conditions, is managed by ZIP14 (SLC39A14) and ZIP8 (SLC39A8) at the plasma membrane. Ferroptosis regulation is broadly categorized into three pathways (Dixon et al., 2012). The glutathione/glutathione peroxidase 4 (GSH/GPX4) pathway, including the inhibition of system Xc^−^, the glutamine pathway, and the p53 regulatory axis (Zhang et al., 2023); Iron metabolism regulation, involving pathways such as the autophagy-related gene 5/7 - nuclear receptor coactivator 4 (ATG5/7-NCOA4) pathway, and iron regulatory element binding protein 2 (IREB2) related to ferritin metabolism, with increased of intracellular Fe^2+^ initiating the Fenton reaction (Zheng and Conrad, 2020); Lipid metabolism-related pathways, where long-chain acyl-CoA synthetase 4 (ACSL4) and lysophosphatidylcholine acyltransferase 3 (LPCAT3) paly roles in lipid regulation and ferroptosis. Additionally, Erastin induces ferroptosis by acting on mitochondria.

Iron is a vital micronutrient essential for numerous physiological processes, with its deficiency and excess profoundly impacting normal bodily functions. Iron overload is a hallmark of ferroptosis. After intestinal absorption, Fe^2+^ is oxidized to Fe^3+^, which is then recognized by the transferrin receptor (TFR) after binding to intracellular transferrin (TF). This complex is endocytosed into cells where six-transmembrane epithelial antigens of the prostate 3 (STEAP3) reduce Fe^3+^ back to Fe^2+^ (Frazer and Anderson, 2014). Fe^2+^ is subsequently incorporated into various iron-binding complexes, facilitating normal physiological reactions. Proper cellular iron transport and regulation are essential for maintaining the body’s internal environment (Figure 1). Iron serve as a redox-active cofactor in numerous biological processes, including respiration, central metabolism, photosynthesis, and the maintenance of cellular redox status. Iron-based sensors function as molecular switches, modulating protein activity in response to changes in cellular redox conditions (Outten and Theil, 2009). When iron-binding complexes reach saturation, excess Fe^2+^ is transported to the labile iron pool (LIP) *via* divalent metal transporter 1 (DMT1) to catalyze further reactions. Fe^3+^ is reduced to Fe^2+^ during transfer from extracellular to intracellular, generating ROS, such as hydroxyl radicals. The accumulation of ROS leads to lipid peroxidation within cell membranes, ultimately causing cell death (Dixon et al., 2012). In adult mammals, iron is absorbed through the duodenum, while embryos acquire iron *via* placental transport. Excess Fe^2+^ is oxidized to Fe^3+^ by ferroportin (FPN), located on the basal surface of the trophoblast layer in the placental interface, indicating its role in transporting iron from maternal circulation to the fetus (Donovan et al., 2000). Ferrostatin-1 (Fer-1) was identified as a potent ferroptosis inhibitor through high-throughput screening of small molecule libraries (Dixon et al., 2012). The anti-ferroptotic effect of Fer-1 is attributed to its ability to eliminate alkoxyl radicals generated by ferrous iron from lipid hydroperoxides, as well as other rearrangement products (Miotto et al., 2020).

$${\text{Fe}}^{2+}+H_{2}O_{2}\to {\text{Fe}}^{3+}+\cdot \text{OH}+{\text{OH}}^{-}$$

$${\text{Fe}}^{3+}+H_{2}O_{2}\to {\text{Fe}}^{2+}+\cdot \text{OOH}+H^{+}$$

$$2H_{2}O_{2}\to \cdot \text{OH}+\cdot \text{OOH}+H_{2}O$$

Iron ions enter cells via TFR, a process that can be inhibited by the expression of heat shock protein beta-1 (HSPB1), which reduces iron uptake and thereby suppress erastin-induced ferroptosis (Sun et al., 2015). Free iron within the cell can be incorporated into iron-binding proteins, such as ferritin, which serve as a storage form of iron. In conditions of iron deficiency, tristetraprolin is expressed to degrade mRNA transcripts, thereby reducing the synthesis of iron-binding proteins and maintaining the capacity of the intracellular iron pool. Ferritin, composed of two subunits—ferritin heavy chain (FTH) and ferritin light chain (FTL)—regulates intracellular Fe^2+^ metabolism. Fe^3+^ that is not reduced to Fe^2+^ by DMT1 is stored in ferritin and can be converted to Fe^2+^ as needed, playing a role in the regulation of ferroptosis and other cellular processes (Torti and Torti, 2013). Post-transcriptional inhibition of ferritin expression by iron-regulatory proteins 1 (IRP1, also known as ACO1) and 2 (IRP2, also known as IREB2), along with an increase in TFR1 levels, enhances cellular sensitivity to ferroptosis. Conversely, nuclear receptor coactivator 4 (NCOA4) mediates selective autophagy, leading to the lysosomal degradation of ferritin and the release of free iron, thereby promoting ferroptosis (Hou et al., 2016). Nuclear factor erythroid 2 related factor 2 (Nrf2), a key regulator of the antioxidant response, when inhibited, leads to the inactivation of GPX4 and FTH, as well as an increase in LIP, which induces ferroptosis (Tao et al., 2022; Anandhan et al., 2023). Nrf2-regulated heme oxygenase-1 (HO-1) is a key mediator of BAY-induced ferroptosis catalyzing heme degradation to produce Fe^2+^ (Chang et al., 2018). In mammalian cells, poly (rC)-binding protein 1 (PCBP1) functions as a cytosolic iron chaperone, binding and delivering iron to recipient proteins. In the absense of PCBP1, iron can generate ROS, leading to lipid peroxidation and triggering ferroptosis. Therefore, PCBP1’s role as an iron chaperone is essential in limiting cytosolic iron toxicity and preventing ferroptosis (Protchenko et al., 2021). Additionly, the transport of iron and ferritin out of the cell is facilitated by ferroportin and the ferritin transfer protein Prominin2, thereby inhibiting ferroptosis (Brown et al., 2019).

The balance of iron within the cell is a delicate equilibrium. Its proper management is crucial for health, and its disruption can have severe consequences. The study of ferroptosis provides valuable insights into this balance and may lead to breakthroughs in treating iron-related disorders. As our knowledge expands, so does the potential to harness ferroptosis for therapeutic benefit, offering hope for the mitigation of iron-induced pathologies.

#### 2.2.2 Lipids metabolism

Polyunsaturated fatty acids (PUFAs), the unsaturated components of the lipid bilayer, play a pivotal role in determining cellular sensitivity to ferroptosis. Placental lipids are composed of approximately 60% phospholipid, 34% cholesterol, 4% cholesterol esters, and 2% triacylglycerol. Free PUFAs must undergo esterification to membrane phospholipids and subsequent oxidation to propagate the ferroptotic (Brown et al., 2016). The weak C-H bonds in the methylene groups adjacent to the C-C double bonds of PUFAs result in a rapid rate of lipid autoxidation. The presence of multiple unsaturations further complicates the product profiles observed in these autoxidations processes, with some intermediate peroxyl radicals undergoing cyclization to form more complex structures (Conrad and Pratt, 2019).

Lipid peroxidation primarily involves non-enzymatic autoxidation and enzymatic processes. Autoxidation refers to the free radical oxidation of organic compounds, which, when involving biologically significant moleculaes like lipids, is termed peroxidation (Yin et al., 2011). Iron can catalyze non-enzymatic lipid autoxidation *via* the Fenton reaction, where free iron reacts with hydrogen peroxide to generate ferrous and hydroxyl radicals. These hydroxyl radicals react with hydrogen atoms at the diallyl position of polyunsaturated fatty acids-containing phospholipids (PUFA-PLs), producing carbon-centered phospholipid radicals (PL•). These radicals then react with oxygen molecules to form phospholipid peroxide radicals (PL-OO•) which in turn extract hydrogen from other PUFAs, leading to the formation of phospholipid hydroperoxide (PL-OOH) and lipid free radicals (PL•), thereby initiating a chain reaction of lipid peroxidation. Enzymatic lipid peroxidation is primarily mediated by lipoxygenases (LOXs) and non-heme dioxygenases (Haeggström and Funk, 2011), with cyclooxygenases (COXs) playing a lesser role (Rouzer and Marnett, 2003). Recently, Fas-associated factor 1 (FAF1) was identified as assembling into a globular structure that sequesters free PUFAs within a hydrophobic core, thus preventing their peroxidation (Cui et al., 2022).

The LOX-catalyzed oxidation of arachidonic acid (AA) and adrenic acid (AdA) can enhance cellular sensitivity to ferroptosis. These key PUFAs are central to lipid peroxidation, a hallmark of ferroptosis in placental trophoblast cells. The formation of AA/AdA-CoA derivatives begins with the ligation of CoA to free AA/AdA by Acyl-CoA synthetase lysophosphatidylcholine acyltransferase 3 (LPCAT3) (Kagan et al., 2017), followed by LPCAT3-mediated esterification of these derivations to AA- and AdA-containing phosphatidylethanolamine (AA-PE and AdA-PE) (Hishikawa et al., 2008), ultimately leading to LOX-catalyzed intracellular ferroptosis. Wenzel et al. found that 15-LOX can bind to phosphatidylethanolamine-binding protein 1 (PEBP1) to generate hydroperoxy-PE, resulting in GPX4 dysfunction and the promotion of ferroptosis (Wenzel et al., 2017). Membrane association induces a conformational change in the 15-LOX/PEBP1 complex, catalyzing the production of pro-ferroptotic death signals (Manivarma et al., 2023). While LOX contributes to early lipid peroxidation in ferroptosis, its inhibition does not prevent cell death, whereas free radical-trapping antioxidants do, indicating that autoxidation is the primary driver of ferroptosis (Shah et al., 2018). Recently, Zou et al. used genome-wide CRISPR/Cas9 suppressor screens to identify cytochrome P450 oxidoreductase (POR) as a key mediator of ferroptotic cell death in cells predisposed to ferroptosis (Zou et al., 2020).

ROS are byproducts of aerobic metabolism in cells, typically neutralized by the cellular antioxidant system. However, excessive ROS can overwhelm this system, leading to the destruction of cellular components and resulting in cell necrosis when antioxidant defenses fail (Niki, 2009). GSH, a small molecule antioxidant, plays a pivotal role in scavenging intracellular ROS. Composed of glutamate, glycine, and cysteine, GSH functions by exchanging disulfide bonds with peroxisomal proteins and serving as a reductant for GSH peroxidase. Cysteine is the rate-limiting amino acid in GSH synthesis (Lu, 2009). Nrf2, the primary transcription factor regulating GSH metabolism, induces the transcription of several key enzymes involved in GSH synthesis and recycling, including glutamate cysteine ligase (GCL), GSH synthase, GPX2, GSH S-transferases (GSTs), and GSH reductase (GR) (Harvey et al., 2009). Among the seven GPXs in mammals, GPX4 is unique in its ability to oxidize GSH to GSSG while reducing cytotoxic lipid peroxides (L-OOH) to their corresponding alcohols (L-OH), preventing ROS formation (Friedmann Angeli and Conrad, 2018).

Acyl-CoA synthetase long-chain family (ACSL4) and LPCAT3 are critical in the oxidation of AA/AdA. ACSL4 facilitates the ligation of free PUFAs with coenzyme A to form PUFA-CoAs, which are essential in PUFA-PLs biosynthesis (Gan, 2022). ACSL4 esterifies coenzyme A to free fatty acids in an ATP-dependent process, activating the fatty acids for subsequent oxidation (Doll et al., 2017). The activation of ACSL4 promotes ferroptosis (Li et al., 2019), while reduced expression of ACSL4 and LPCAT3 decreases lipid peroxide accumulation, thus inhibiting ferroptosis. LOXs also contribute to PUFA oxidation by catalyzing the addition of oxygen atoms to the double bonds of PUFAs, leading to lipid peroxidation. Inhibition of LOXs has been shown to suppress ferroptosis (Shah et al., 2018). Magtanong et al. demonstrated that exogenous monounsaturated fatty acids (MUFAs) can mitigate the effects of system Xc^−^ inhibition, cystine deprivation, or GPX4 inactivation, effectively preventing the iron-dependent oxidative cell death characteristic of ferroptosis, particularly in the presence of ACSL3 (Magtanong et al., 2019). Stearoyl-CoA desaturase-1 (SCD1), which catalyzes the conversion of saturated fatty acids to MUFAs, plays a critical role in this process. Inhibition of SCD1 reduces CoQ10 levels, thereby inducing cellular ferroptosis (Tesfay et al., 2019).

The intricate interplay between PUFAs, lipid peroxidation, and ferroptosis reveals the complex regulatory mechanisms underlying cellular redox homeostasis. The sensitivity of PUFAs to oxidation and their role in ferroptosis highlight the potential for targeted therapies in diseases characterized by lipid metabolism dysregulation. The delicate balance between iron, lipid metabolism, and cellular antioxidant systems is crucial for maintaining cellular integrity and preventing ferroptosis. As research continues to unravel the complexities of ferroptosis, the potential for developing targeted therapies to modulate this form of cell death becomes increasingly promising.

#### 2.2.3 The system Xc^−^-GSH-GPX4 pathway

**FIGURE 2:**
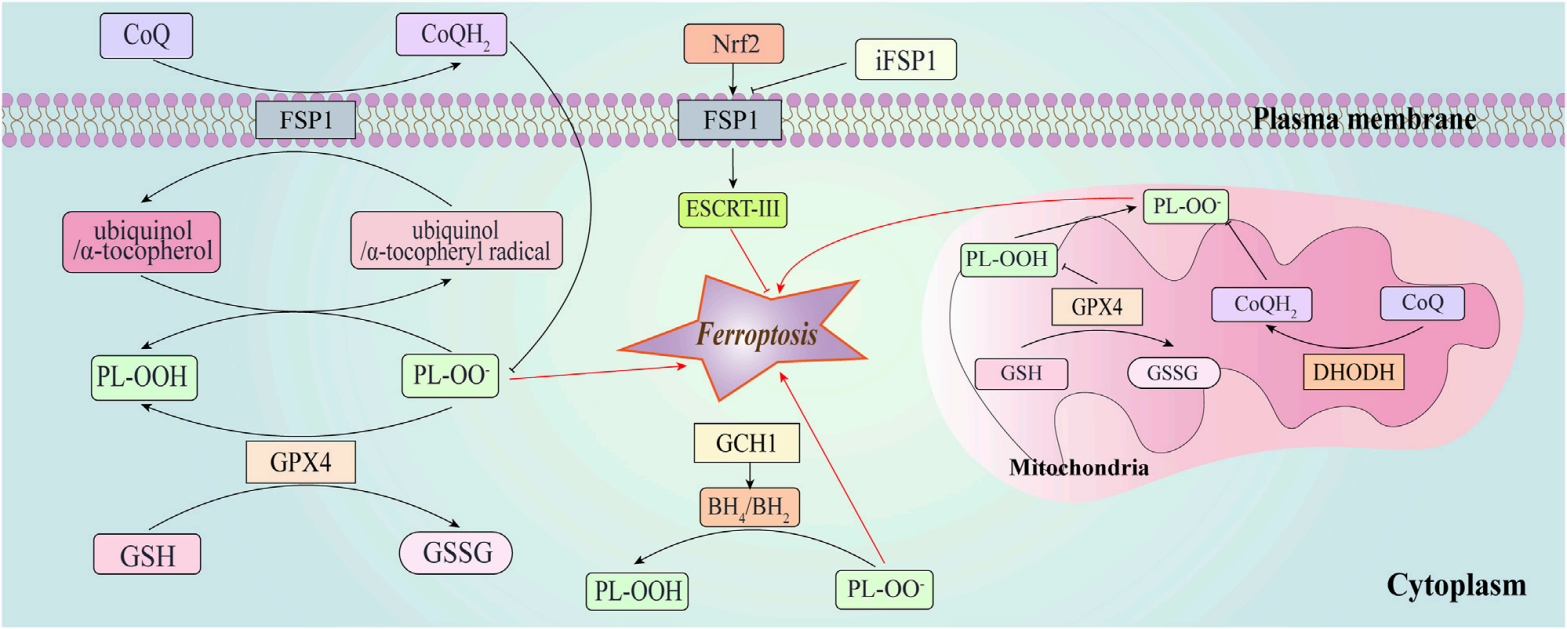
Ferroptosis-suppressing pathways. This figure depicts GPX4, ferroptosis suppressor protein 1 (FSP1), dihydroorotate dehydrogenase (DHODH), and GTP cyclohydrolase-1 (GCH1) suppress ferroptosis at different subcellular compartments. FSP1 is located at the plasma membrane where it functions as an oxidoreductase that reduces coenzyme Q10 (CoQ), suppressing lipid peroxidation. It prevents lipid peroxidation and associated ferroptosis via the reduction of ubiquinol/α-tocopherol on the level of lipid radicals unlike GPX4/GSH. FSP1 can also promote the endosomal sorting complex required for transport (ESCRT)-III to enhance the repair of cell membranes in a CoQ10-independent manner. FSP1-CoQ10-NAD(P)H axis can be activated through nuclear factor erythroid 2 related factor 2 (Nrf2) to inhibit ferroptosis. Synthesis of tetrahydrobiopterin/dihydrobiopterin (BH4/BH2) by GCH1-expressing cells caused lipid remodeling, suppressing ferroptosis by selectively preventing depletion of phospholipids.

Several pathways are involved in suppressing ferroptosis (Figure 2). System Xc^−^ is a cystine/glutamate antiporter protein that is widely distributed in the phospholipid bilayer of cell membranes and consists of two subunits, solute carrier family members (SLC7A11 and SLC3A2) (Lewerenz et al., 2013). Due to the rapid reduction of intracellular cystine, the intracellular concentration of cysteine is typically higher than extracellular levels. System Xc^−^ imports cystine and exports glutamate (Bannai, 1986), with the imported cystine participating in the synthesis of GSH. Deletion of SLC7A11 induces tumor-selective ferroptosis and inhibits the growth of pancreatic ductal adenocarcinoma (PDAC) growth (Badgley et al., 2020).

P53 inhibits cystine uptake and sensitizes cells to ferroptosis by suppressing SLC7A11 expression (Jiang L. et al., 2015). Interferon γ (IFNγ), released by CD8 T cells, downregulates the expression of SLC3A2 and SLC7A11, promoting lipid peroxidation and ferroptosis in tumor cells (Wang W. et al., 2019). Chu et al. found that SLC7A11 specifically binds to and inhibits the enzymatic activity of arachidonate 12-lipoxygenase (ALOX12). Inactivation of ALOX12 impedes p53-mediated ferroptosis induced by oxidative stress and removes the suppression of p53-dependent tumor growth. Notably, ALOX12 does not significantly affect ferroptosis triggered by erastin or GPX4 inhibitors (Chu et al., 2019). Erastin induces ferroptosis by inhibiting system Xc^−^, thereby impairing cysteine entry into cells and inhibiting GSH synthesis, leading to decreased GPX activity and ROS accumulation (Dixon et al., 2012). Erastin also induces mitochondrial dysfunction *via* VDACs and can negatively regulate ferroptosis by stimulating heat shock protein beta-1 (HSPB1) expression, reducing cellular iron uptake and lipid ROS production (Sun et al., 2015).

GPX4 reduces phospholipid hydroperoxide products (AA/AdA-PE-OOH) to their corresponding phosphatidyl alcohols (PLOH). As a key inhibitor of ferroptosis, the loss of GPX4 activity leads to the accumulation of lipid peroxides, a hallmark of ferroptosis (Forcina and Dixon, 2019). GPX4, being a selenoprotein, requires selenocysteine for its biosynthesis, and selenium is critical for its anti-ferroptotic effects (Friedmann Angeli and Conrad, 2018). At the transcriptional level, gpx4 can produce three different proteins-mitochondrial (m-), nuclear (n-), and cytosolic (c-) GPX4 due to the presence of multiple transcriptional start sites, but only c-GPX4 is essential for ferroptosis (Liang et al., 2009). The expression of GPX4 is regulated by transcription factors such as stimulating protein 1 (SP1), nuclear factor Y (NF-Y), and activator protein (AP) (Ufer et al., 2003). Seiler et al. induced GPX4 inactivation in mice and cells, finding that 12/15-LOX knockout cells were unaffected by GSH deprivation, suggesting that cell death induced by GSH depletion or reduced GPX4 activity requires 12/15-LOX (Seiler et al., 2008). The translocation of apoptosis-inducing factor (AIF) from mitochondria to the nucleus was also observed, indicating that GPX4 inactivation can trigger AIF-mediated cell death (Seiler et al., 2008). Yang and colleagues identified the first GPX4 inhibitor, RAS-selective lethal compounds 3 (RSL-3), which, unlike erastin, does not act on system Xc^−^, but directly inhibits GPX4, triggering ferroptosis without depletion of GSH (Yang et al., 2014). Treatment of RSL3-exposed primary human renal proximal tubule epithelial cells (HRPTEpiCs) with liproxstatin-1 (Lip-1) has shown that Lip-1 protects against RSL3-induced cell death, and Lip-1 can inhibit RSL3-induced oxidation of BODIPY 581/591 C11 (Friedmann Angeli et al., 2014).

Dihydroorotate dehydrogenase (DHODH) is an enzyme situated on the outer surface of the mitochondrial inner membrane, the inactivation of which induces extensive mitochondrial lipid peroxidation and ferroptosis in cancer cells with low GPX4 expression (Mao et al., 2021). DHODH functions in parallel with mitochondrial GPX4 but operates independently of cytoplasmic GPX4, to prevent ferroptosis of the inner mitochondrial membrane. It achieve this by reducing ubiquinone to ubiquinol, a free-radical-trapping antioxidant that possesses anti-ferroptotic properties (Mao et al., 2021).

The intricate regulatory network controlling ferroptosis underscores the complexity of cellular redox balance. The interplay between ferroptosis and other cellular processes, such as mitochondrial function and amino acid transport, suggests that ferroptosis is not an isolated event but rather deeply integrated into cellular physiology. The role of GPX4 and its dependence on selenium highlight the potential for dietary or supplemental interventions to modulate susceptibility to ferroptosis. This could have implications for the prevention or treatment of diseases associated with oxidative stress, including neurodegenerative disorders and certain cancers.

#### 2.2.4 The FSP1-CoQ10-NAD(P)H system

Recent studies have identified ferroptosis suppressor protein 1 (FSP1) as an inhibitor of ferroptosis that operates independently of the system Xc^−^-GSH-GPX4 axis (Doll et al., 2019; Bersuker et al., 2019). FSP1 was discovered as a significant factor in ferroptosis resistance through a synthetic lethal CRISPR-Cas9 screen following RSL3 treatment (Bersuker et al., 2019). Coenzyme Q10 (CoQ10), a key component of the mevalonate pathway, is the only endogenously synthesized lipid-soluble antioxidant present in all membranes (Bentinger et al., 2007; Kaymak et al., 2020). A reduction in CoQ10 levels increases cellular susceptibility to ferroptosis; one study found that FIN56 depletes CoQ10 and promotes ferroptosis by regulating the mevalonate pathway (Shimada et al., 2016).

FSP1, initially recognized as a response gene to the oncogene p53 (Horikoshi et al., 1999), functions as an oxidoreductase of CoQ, catalyzing the regeneration of CoQ10 *via* NAD(P)H in the plasma membrane. This process produces ubiquinol, which directly reduces lipid radicals, thereby terminating lipid autoxidation and preventing the accumulation of lipid peroxides (Yan et al., 2021; Marshall et al., 2005). FSP1 is capable of protecting cells from ferroptosis induced by GPX4 deficiency. The absence of either GPX4 or FSP1 leads to the accumulation of lipid peroxides in the cells, triggering ferroptosis, which suggests a synergistic inhibitory role of FSP1 and GPX4 in ferroptosis (Doll et al., 2019; Plays et al., 2021). Additionally, Mishima et al. discovered that FSP1 also functions as a vitamin K reductase, effectively reducing vitamin K to its hydroquinone form. This form acts as a potent radical-trapping antioxidant and inhibitor of lipid peroxidation, thereby further inhibiting ferroptosis. This mechanism explains vitamin K’s role in counteracting the toxic effects of warfarin (Mishima et al., 2022). FSP1 can prevent erastin- and RSL3-induced ferroptosis through a ubiquinol-independent mechanism. Specifically, FSP1-dependent recruitment of endosomal sorting complexes required for transport (ESCRT)-III facilitates resistance to ferroptosis by activating a membrane repair mechanism that regulates membrane outgrowth and fission (Dai et al., 2020; Li et al., 2023). Moreover, the regulation of FSP1 involves cAMP-response-element-binding protein and Nrf2 (Nguyen et al., 2020; Chorley et al., 2012). The negative regulators of p53, MDM2 and MDMX, also influence FSP1 by modulating the peroxisome proliferator-activated receptor-α (PPARα) (Venkatesh et al., 2020).

The discovery of FSP1 as a distinct ferroptosis suppressor highlights the complexity of cellular mechanisms that govern this form of cell death. FSP1’s multifaceted role in ferroptosis, through its interaction with CoQ10 and its independent mechanism from the GSH-GPX4 axis, suggests that it could be a key target for therapeutic interventions in diseases where ferroptosis plays a significant role.

#### 2.2.5 The GCH1/BH4/DHFR system

Kraft et al. identified a sset of genes that conteract cellular ferroptosis independently of the GPX4/GSH system, among which are GTP cyclohydrolase-1 (GCH1) and its metabolic derivatives tetrahydrobiopterin/dihydrobiopterin (BH4/BH2) (Kraft et al., 2020). Overexpression of GCH1, MS4A15, and OLFR367-ps not only reduces lipid peroxidation but provides nearly complete protection against ferroptosis (Kraft et al., 2020). BH4 serves as a potent free-radical-trapping antioxidant, capable of directly decreasing the accumulation of PUFA-containing lipid peroxides, thereby shielding cells from lipid peroxidation and ferroptosis, as well as contributing to the synthesis of CoQ10. Inhibition of dihydrofolate reductase (DHFR), which catalyzes the regeneration of BH4, using methotrexate can synergize with GPX4 inhibition to induce ferroptosis (Soula et al., 2020). BH4 synthesis supports cell proliferation even in the presence of GPX4 inhibition induced by RSL3 and ML21, yet it proves ineffective against erastin-induced GPX4 inhibition, highlighting that the role of BH4 independently of cystine depletion (Soula et al., 2020). GCH1 has also been recognized as a polymorphic locus linked to pain sensitivity, cardiovascular risk, and chronic diseases such as diabetes (Tegeder et al., 2006; Takimoto et al., 2005; Alp et al., 2003). Recently, Cronin et al. demonstrated that genetic inactivation of GCH1 significantly impairs T-cell proliferation in mature mice and humans, while enhancement of BH4 levels *via* GCH1 overexpression boosts the response of CD4^+^ and CD8^+^ T-cells, thereby enhancing their anti-tumor activity (Cronin et al., 2018).

The identification of GCH1 and BH4 as key regulators of ferroptosis provides a deeper understanding of the complex interplay between cellular metabolism, redox balance, and cell death pathways. The ability of BH4 to directly reduce lipid peroxides and its role in CoQ10 synthesis underscore its dual function as an antioxidant and a modulator of cellular bioenergetics. the multifaceted role of GCH1 and BH4 in regulating ferroptosis, immune function, and cellular metabolism highlights their potential as therapeutic targets. Further research into the mechanisms by which these molecules modulate ferroptosis could lead to the development of new treatments for a range of diseases.

### 2.3 Ferroptosis inducers and inhibitors

Numerous genes and signaling pathways are involved in both the induction and inhibition of ferroptosis, offering new avenues for potential therapeutic interventions in related diseases (Tables 2, 3).

**TABLE 2:** Ferroptosis inducers (FINS).

Class	Compounds or drugs	Mechanism
I	Erastin and its derivatives	Inhibit system Xc^−^ and prevent cystine import
	Sorafenib	Inhibit system Xc^-^
	Sulfasalazine	Prevent cystine import
	Acetaminophen	Consume intracellular GSH
	L-buthionine-(S,R)-sulfoximine	Block the biosynthesis of GSH
II	RSL3	Bind GPX4 at the selenocysteine active site
	ML162	
	ML210	
	Cisplatin	Bind GSH and inactivate GPX4
	FIN56	Induce GPX4 degradation
	DPI10	Inhibit GPX4 activity
III	iFSP1	Inhibit FSP1 activity and reduce CoQ10 production
	Statin	Inhibit the mevalonate pathway
IV	FINO2	Oxidize Fe^2+^, promote ROS accumulation and oxidize PUFA
	Heme	Increase the intracellular unstable iron
	Artemisinin and its derivatives	Induce ferritin autophagy to release unstable iron
	Lapatinib	Induce mitochondrial dysfunction to enhance oxidative stress and ferroptosis

**TABLE 3:** Ferroptosis inhibitors.

Class	Compounds or drugs	Mechanism
Iron chelators	Deferiprone	Reduce intracellular iron and inhibit Fenton reaction
	Deferoxamine	
	Deferasirox	
	Hepcidin	
	Curcumin	
Antioxidants	Ferrostatin-1	Remove ROS and reduce intracellular unstable iron
	Liproxstatin-1	Remove ROS and activate the Nrf2 pathway
	Trolox	Inhibit lipid peroxidation
	XJB-5–131	Increase the expressions of GPX4
	1,25(OH)_2_D_3_	
	Selenium	Enhance the number of selenoproteins
LOX inhibitors	Zileuton	Inhibit 5-LOX
	PD-146176	inhibit 15-LOX
	Vitamin E	
	Baicalein	inhibit 12/15-LOX
ACSL4 inhibitors	Rosiglitazone	Inhibit ACSL4 function and block the PUFA activation
	Pioglitazone	

## 3 Ferroptosis in gestation

### 3.1 Gestational physiology

Iron is essential for fetal growth and development, with the placenta acting as an intermediary to facilitate the exchange of materials between mother and fetus, enabling the fetus to obtain iron from maternal circulation. The maternal dietary intake of ferric iron (Fe^3+^), with 85%–90% absorbed as non-heme iron, serves as a primary source of iron for fetal development (Raper et al., 1984). Fe^3+^ is reduced to ferrous iron (Fe^2+^) by reductases such as duodenal cytochrome b reductase 1 (DCYTB), prion-protein (PrPC), and Steap3 (McKie et al., 2001; Ohgami et al., 2005; Tripathi et al., 2015), which are then absorbed through DMT1 located at the apical part of enterocytes.

The ferrous oxidase hephaestin (Heph) and FPN in the intestinal tract facilitate the absorption of iron into the systematic circulation (Yeh et al., 2011). Otherwise, iron binds to ferritin and is excreted with intestinal cells. Heme iron absorption and nutrient transport occur primarily through the syncytiotrophoblast, with these cells expressing transporter proteins that mediate iron transfer. Iron is primarily transported from the maternal circulation to the placenta bound to transferrin, ferritin, or heme, with transferrin-bound iron (TBI) being the predominant form absorbed by the placenta (Sangkhae and Nemeth, 2019). TBI is transferred to the apical side of the placental trophoblast *via* TFR1. Knockout of TFR1 in mice, resulting in a deficiency in both the embryo and placenta, leads to severe anemia and high embryonic lethality (Levy et al., 1999). DMT1, located in the cytoplasm and basement membrane of the syncytiotrophoblast, mediates the movement of ferrous ions from endosomes to the cytoplasm and across the basement membrane (Chong et al., 2005). In the case of iron overload, when iron is overloaded, transferrin is saturated, excess iron forms non-transferrin-bound iron (NTBI) in the circulation. It has been discovered that in addition to DMT1, endosomal transport proteins ZIP14 (SLC39A14) and ZIP8 (SLC39A8), which mediate NTBI uptake at the plasma membrane, are also present in the placenta (Liuzzi et al., 2006). Iron can be stored in the placenta or transferred into the fetal matrix *via* FPN, and then cross the endothelium to enter the fetal circulation as NTBI (Evans et al., 2011). Complete knockout of FPN, results in embryonic lethality, whereas conditional knockout that preserves FPN, expression in the placenta allows normal embryonic development, underscoring the critical role of FPN in placental iron export (Donovan et al., 2005).

During the first trimester, iron requirements decrease compared to pre-pregnancy levels due to the cessation of menstruation. However, as pregnancy advances, maternal RBC mass increases, and placental and fetal growth accelerates, leading to a heightened physiological demand for iron in the later stage of pregnancy (Fisher and Nemeth, 2017). The placenta retains approximately 90 mg of iron to maintain its function and delivers around 270 mg to the fetus over the course of the pregnancy (Cao and O'Brien, 2013). The expansion of maternal plasma volume and increasing need for iron to support fetal growth during the second and third trimesters result in elevated erythropoiesis to sustain hemoglobin levels, ultimately depleting maternal iron reserves. An estimated 36% of pregnant women aged 15–49 globally suffer from anemia with around 40% of these cases attributed to iron deficiency (Ataide et al., 2023). Severe anemia is associated with adverse outcomes such as *postpartum* hemorrhage, maternal mortality, and long-term complications, including low birth weight and neonatal growth retardation (Daru et al., 2018).

Both iron deficiency and iron overload can pose risks, creating a U-shaped relationship between maternal hemoglobin concentration and the risk of adverse pregnancy outcomes (Dewey and Oaks, 2017). Iron supplementation to pregnant women with sufficient iron stores may also present potential risks (Georgieff et al., 2019). In pathological states where large amounts of iron are suddenly released from damaged cells, NTBI can react with oxygen to form ROS, which can damage cellular lipid membranes, leading to oxidative stress and the activation of programmed cell death pathways such as ferroptosis and iron-associated autophagy (Tang et al., 2021). High iron levels are also associated with increased blood viscosity and a diminished systemic response to inflammation and infection. Serum ferritin serves as a biomarker for maternal iron status. Studies indicate that higher ferritin concentrations in early pregnancy are linked to more favorable pregnancy outcomes, whereas high ferritin levels in late pregnancy are associated with poorer outcomes, including preterm labor and low birth weight (Dewey and Oaks, 2017; Chang et al., 2003; Hanieh et al., 2013). Research conducted in developing countries has shown that high doses of in-home iron fortification can lead to iron accumulation in the colon, altering the infant gut microbiome and causing intestinal inflammation (Jaeggi et al., 2015).

Neonates preferentially absorb heme iron of animal origin ingested by the mother during pregnancy, as opposed to maternal iron intake in the form of ferrous sulfate (Young et al., 2012). Thus, pregnant woman require additional dietary iron to meet the increasing demands as gestation progresses. Hepcidin, the primary regulator of systemic iron homeostasis, controls plasma iron concentration and the distribution of iron in tissues by inhibiting intestinal iron absorption, iron recycling in macrophages, and iron mobilization from hepatic stores (Nemeth and Ganz, 2006). During normal pregnancy, maternal hepcidin levels are significantly reduced to facilitat sufficient iron transport across the placenta. Recently Sangkhae et al. discovered that maternal hepcidin levels play a pivotal role in determining embryonic and fetal iron endowment, with higher hepcidin levels potentially leading to embryonic anemia and increased embryonic mortality (Sangkhae et al., 2020). The gradual decrease in hepcidin levels from early to late gestation enhances iron release from enterocytes and hepatocytes, consequently elevating iron levels in the placenta (van Santen et al., 2013). Yang et al. found that estrogens inhibit the hepcidin transcription in hepatocytes *in vivo* and *in vitro* in mice, thereby increasing iron uptake (Yang et al., 2012).

A study utilizing single-cell transcriptomics was conducted to explore the cell-cell interactome between fetal placental trophoblast cells and maternal endometrial stromal cells (Pavličev et al., 2017). Wu et al. analyzed this dataset with a particular focus on genes related to iron homeostasis and ferroptosis, concluding that ferritin genes are highly expressed across major cell types. Additionally, they found that trophoblast cells might be more susceptible to ferroptosis compared to other cell types at the maternal-fetal interface due to the high expression of Lpcat3 and Sat1, both of which play critical roles in ferroptosis (Ng et al., 2019).

**FIGURE 3:**
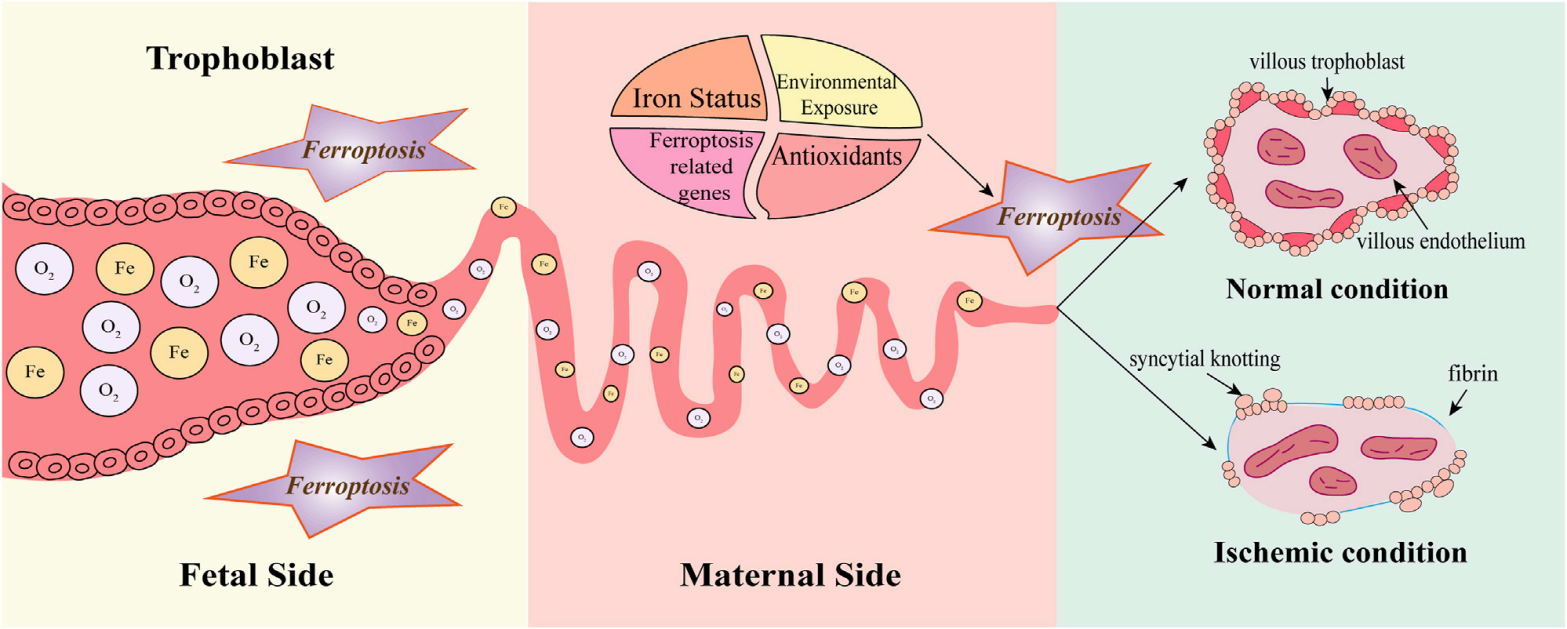
Ferroptosis in early gestation. Before 8–10 weeks, spiral arteries are completely blocked by endothelial cells and clots, creating hypoxia and hypoglycemia in the embryo. Until the end of the first trimester (10–12 weeks), the spiral arteries open and a sudden increase in oxygen and iron can significantly accumulate ROS, oxidative stress, and tissue damage, inducing ferroptosis which contributes to remodeling the spiral arteries. Normally, villi are covered with villous cytotrophoblast and syncytiotrophoblast, containing fetal vessels covered with villous endothelium. Extravillous trophoblast (EVT) invasion causes spiral artery remodeling thereby creating low-resistant perfusion. Under ischemic conditions, the villous trophoblast becomes discontinuous. Increased fibrin and syncytial knotting deposit on the villous surface. Spiral arteries are inappropriately remodeled due to impaired EVT invasion and ischemia exposes villous tissue to increased oxidative stress. Factors such as iron status, antioxidants, environmental exposure, and ferroptosis-related genes can influence the process. However, the persistence of ferroptosis may cause pathological effects such as shallow trophoblast invasion and narrowing of the vascular lumen.

The placental bed is composed of uterine spiral arteries that deliver oxygen-rich blood to the developing fetus and placenta. Until 8–10 weeks of gestation, these maternal spiral arteries remain obstructed by congealed endothelial cells and blood clots, creating a hypoxic and hypoglycemic environment for the embryo (Ng et al., 2019). At 10–12 weeks of gestation, the spiral arteries become canalized, allowing maternal blood to flood into the placental lacunae, and exposing the fetal villi to a nutrient-rich environment containing glucose, oxygen, and iron (Ng et al., 2019). This process is kin to a hypoxia/reperfusion event, which can lead to significant oxidative stress and tissue injury. Spiral artery remodeling begins after blastocyst implantation with the invasion of extravillous trophoblast (EVT) cells into the decidua and the formation of a continuous EVT shell at the maternoplacental interface (Burton et al., 1999). Incomplete occlusion and fragmentation of the EVT shell due to insufficient EVT cells invasion into the spiral arteries, result in a relatively hyperoxic environment, potentially leading to the generation of ROS (Hempstock et al., 2003). An imbalance between oxidant and antioxidant activities, known as oxidative stress, is driven by increased ROS, which induces ferroptosis and contributes to the remodeling of spiral arteries (Figure 3). However, excessive and prolonged oxidative stress can lead to persistent ferroptosis, resulting in pathological outcomes such as shallow trophoblast invasion and vessel luminal narrowing (Gumilar et al., 2023).

### 3.2 Pre-eclampsia (PE)

PE is a pregnancy disorder, typically manifesting after the 20th week of gestation, characterized by hypertension and proteinuria. Oxidative stress is believed to induce lipid peroxidation of cell membranes and excessive ferroptosis at the maternal-fetal interface leading to the pathological features of PE, including shallow endovascular invasion of EVT cells and inadequate remodeling of maternal spiral arteries.

The GPX4 gene variant (rs713041), linked to oxidative stress, has been associated with increased susceptibility to PE in the Chinese Han population (Chen et al., 2020). Selenium, essential for GPX4 function, plays a critical role in inhibiting ferroptosis (Ingold et al., 2018). Min et al. demonstrated an inverse relationship between blood selenium levels and the risk of PE (Xu et al., 2016). Research has consistently shown that serum iron concentration, ferritin levels, and percent transferrin saturation are significantly eleveted in patients with PE compared to normal pregnancies (Rayman et al., 2002; Gutierrez-Aguirre et al., 2017).

In PE models, upregulation of miR-30b-5p leads to reduced GSH and elevated labile Fe^2+^, both of which are crucial in promoting ferroptosis (Zhang et al., 2020). This finding suggests that targeting this microRNA could be a therapeutic strategy to mitigate the effects of PE. Additionally, markers of mitochondrial fission/fusion, apoptosis, and mitochondrial complex expression are differentially disrupted in the full-term preeclamptic placenta. The level of ROS signaling in these placentas may be sufficient to trigger compensatory antioxidant and mitochondrial responses, helping maintain tissue function despite the pathological stress (Holland et al., 2018).

The interplay between oxidative stress, ferroptosis, and the dysregulation of iron metabolism in the pathogenesis of PE underscores the complexity of this pregnancy disorder. The identification of specific genetic variants and environmental factors such as selenium deficiency that contribute to the risk of PE highlights potential avenues for early diagnosis and intervention. Moreover, understanding the compensatory mechanisms that maintain tissue function in the face of oxidative stress may lead to the development of novel treatments aimed at bolstering these natural defenses, thereby improving outcomes for both mother and child. Further research is necessary to fully elucidate these mechanisms and to translate these findings into clinical practice.

### 3.3 Gestational diabetes mellitus (GDM)

GDM is traditionally defined as glucose intolerance of varying degrees that develops or is first detected during pregnancy. Hyperglycaemia is believed to induce ferroptosis due to high glucose conditions, decreased glutathione levels, disrupted iron transport, and increased lipid peroxidation in trophoblast cells. The accumulation of iron contributes to elevated lipid peroxidation which subsequently triggers ferroptosis. The role of iron metabolism and oxidative stress in GDM pathology suggests that interventions targeting these pathways could potentially mitigate the development or severity of GDM.

Two decades ago, Lao et al. identified a correlation between elevated serum ferritin levels and glucose intolerance in the third trimester among non-anemic women (Lao et al., 2001). Since then, elevated ferritin levels have been associated with an increased risk of GDM (Sun et al., 2020). The identification of specific biomarkers, such as elevated ferritin and hemoglobin, could improve early detection and allow for more timely interventions. A recent meta-analysis confirmed that patients with GDM exhibit higher levels of serum iron, ferritin, transferrin saturation, hepcidin, and hemoglobin, alongside a lower total iron-binding ability, compared to those without GDM. This suggests that high serum ferritin and hemoglobin levels are linked to an elevated risk of GDM (Yang K. et al., 2022). Monitoring and managing iron levels, as well as antioxidant therapies, might be beneficial in high-risk pregnancies. In trophoblast cells affected by GDM, mitochondria were observed to significantly swollen, with notable changes such as the disappearance or reduction, which are indicative of ferroptosis (Zhao et al., 2023). Additionally, the expression of genes related to ferroptosis is altered in placental tissues of patients with GDM (Zhao et al., 2023).

The connection between GDM and ferroptosis offers a compelling avenue for research into the mechanisms underlying this pregnancy complication. Furthermore, understanding the molecular changes in placental tissues associated with GDM could lead to targeted therapies that protect the placenta from ferroptosis, thereby potentially improving pregnancy outcomes.

It is also important to consider the broader implications of these findings for maternal and fetal health beyond the pregnancy itself. GDM is known to increase the risk of developing type 2 diabetes later in life (Vounzoulaki et al., 2020; Moon and Jang, 2022). Understanding the role of ferroptosis may provide insights into these long-term health consequences. Overall, while more research is needed to fully understand the interplay between GDM and ferroptosis, these findings provide a foundation for developing novel preventive and therapeutic strategies for GDM and its associated complications.

### 3.4 Recurrent pregnancy loss (RPL)

RPL, also known as recurrent spontaneous abortion (RSA), is defined as the occurrence of two or more consecutive miscarriages. Decidual stromal cells (DSCs) are the predominant cell type in the maternal decidua during early pregnancy. In patients with PRL, communication between these stromal cells and other cell types is disrupted, leading to heterogeneity among maternal decidual cells (Du et al., 2021). Lipid metabolism and redox balance are essential to placental health. The phospholipase A2 (PLA2) protein family comprises lipid-modifying enzymes, with PLA2G6 specifically expressed in the human placenta. Trophoblasts deficient in PLA2G6 exhibit increased sensitivity to ferroptosis when GPX4 activity is absent (Beharier et al., 2020). Novel therapeutic strategies may aim at supporting trophoblast function and reducing the risk of miscarriage. For instance, interventions that enhance GPX4 activity or stabilize lipids to prevent peroxidation could be beneficial. Recent research by Peng et al. has indicated that hypoxia can trigger ferroptosis in human trophoblast cells, which may contribute to miscarriage (Tian et al., 2024). It provides a potential mechanistic link between environmental factors and pregnancy loss, as hypoxia is a common feature in various pregnancy complications.

The connection between ferroptosis and RPL suggests that cellular susceptibility to ferroptosis may play a significant role in pregnancy failure. In conclusion, RPL can have significant physical and psychological impacts on affected women, and an improved understanding of the underlying mechanisms could lead to better support and care for those experiencing recurrent miscarriages. While more research is needed to fully elucidate the mechanisms involved in RPL, the emerging link between ferroptosis and RPL offers promising avenues for the development of new diagnostic and therapeutic approaches to improve pregnancy outcomes for women with a history of recurrent miscarriages.

### 3.5 Intrahepatic cholestasis of pregnancy (ICP)

Cholestatic liver disease is classified into intrahepatic cholestasis (IHC) and extrahepatic cholestasis based on the location of the bile flow obstruction. IHC is caused by disorders affecting hepatic parenchymal cells and/or intrahepatic bile ducts, leading to impaired metabolism and transport of bile acids within the liver. In contrast, extrahepatic cholestasis, also known as obstructive cholestasis, arises from obstruction in the extrahepatic bile duct disease. ICP is the most common liver disease during pregnancy, characterized by pruritus and elevated bile acids, typically occurring in the third trimester and resolving quickly after delivery (Geenes and Williamson, 2009). ICP is diagnosed when nonfasting total bile acid levels reach or exceed 19 μmol/L after excluding other potential causes (Girling et al., 2022). Elevated bile acid levels are associated with an increased risk of adverse pregnancy and fetal outcomes (Wikström Shemer et al., 2013). Women with ICP are more likely to experience GDM, PE, and both spontaneous and iatrogenic preterm delivery compared to those without ICP (Wikström Shemer et al., 2013).

Oxidative stress induced by bile acids is thought to contribute to the pathogenesis of ICP (Schoots et al., 2018). Perez et al. found increased serum bile acid concentrations and signs of oxidative stress in the placenta in ICP rat models, and UDCA treatment can partially prevent changes in the antioxidant system (Perez et al., 2006). Studies have shown that blood selenium levels are significantly lower in patients with ICP compared to those with normal pregnancies (Reyes et al., 2000). Similarly, plasma GPX levels are also significantly reduced in patients with ICP (Hu et al., 2015). Patients with ICP exhibit higher superoxide dismutase (SOD) and malondialdehyde (MDA) activity in maternal erythrocytes, and MDA activity in umbilical cord blood is elevated compared to normal pregnancies (Zhu et al., 2019). Fang et al. identified differential gene expression in placenta samples from normal and ICP pregnancies, discovering that epidermal growth factor receptor (EGFR), associated with ferroptosis, could be a potential target for ICP therapy (Fang and Fang, 2022). Nuclear receptors (NR), a family of transcriptional regulators, are activated by binding to bile acids. Farnesoid X receptor (FXR), a member of this family, becomes activated upon binding bile acids (Makishima et al., 1999). Excessive activation of FXR inhibits bile acid production by inducing the expression of small heterodimer partner 1 (SHP-1), which represses the expression of cytochrome P450 7A1 (CYP7A1), a key enzyme in bile acid synthesis (Calkin and Tontonoz, 2012; Goodwin et al., 2000). Therefore, alterations in FXR are implicated in cholestasis. Tschuck et al. found that FXR agonists such as Turofexorate and Fexaramine limit lipid peroxidation and ferroptosis. FXR activation upregulates ferroptosis-inhibitory proteins such as FSP1, PPARα, GPX4, SCD1, and ACSL3, thereby reducing lipid peroxidation (Tschuck et al., 2023). The FXR agonist W450 significantly reduces bile acid levels and induces the expression of bile acid transporters in the placenta, thereby protecting the placenta from damage caused by oxidative stress (Wu et al., 2015). Modulating specific pathways involving potential therapeutic targets, such as EGFR and FXR agonists, could protect the placenta and improve pregnancy outcomes.

Research on ICP placentas also reveals increased susceptibility to acute hypoxia and ischemia-reperfusion injury, with altered expression of hypoxia-regulated genes like hypoxia-inducible factor-1α (HIF-1α), regulated in development and DNA damage response 1 (REDD1), and mammalian target of rapamycin (mTOR) (Wei and Hu, 2014).

The complex interplay between bile acid-induced oxidative stress, ferroptosis, and the placenta’s vulnerability to hypoxia in ICP highlights the multifaceted nature of this pregnancy complication. While more research is needed to fully understand the pathophysiology of ICP, the current findings provide valuable insights into potential therapeutic targets and the importance of placental health in pregnancy. Understanding how alterations in lipid metabolism and oxidative stress contribute to ICP could lead to novel preventive and therapeutic strategies, potentially benefiting both the mother and the fetus.

## 4 Ferriotsis in liver diseases

Cell death is a key driver in the onset and progression of various liver diseases, with viral, toxic, immunologic, or metabolic factors potentially leading to apoptosis, necrosis, and necroptosis of hepatocytes. This cell death results in inflammation and compensatory cell proliferation, processes closely linked to the development of liver fibrosis, cirrhosis, and hepatocellular carcinoma (Luedde et al., 2014; Schwabe and Luedde, 2018). Programmed cell death modalities, including necroptosis, pyroptosis, and ferroptosis, are mechanisms designed to eliminate intracellular pathogens or mutated cancer cells. However, these processes also result in cell rupture, and the release of intracellular immune contents, triggering inflammatory immune responses and potentially harming healthy cells (Kolb et al., 2017). Studies have indicated that the GSH/GPX4 axis is essential in preventing lipid oxidation-induced acute renal failure, and ferroptosis significantly contributes to liver damage and renal tissue injury due to ischemia/reperfusion (Friedmann Angeli et al., 2014).

While cell death is a natural and necessary process, its dysregulation can have severe consequences for liver health. The recognition that ferroptosis plays a significant role in hepatic and renal injury provides a new perspective on the management of liver diseases. Further research into the mechanisms underlying ferroptosis and its impact on liver disease progression is essential for developing effective therapeutic strategies.

### 4.1 Alcoholic liver disease (ALD)

Recent studies have highlighted the significant role of ferroptosis in the development of ALD, a major form of liver damage caused by excessive alcohol consumption. Acute and chronic ethanol exposure contributes to an increase in mitochondrial ROS in the liver, which is associated with a decrease in the activity of the mitochondrial GSH antioxidant system and oxidative damage to mitochondrial proteins and DNA. Meanwhile, iron overload, as a key inducer of lipid accumulation and ferroptosis, is related to the progression and mortality of ALD (Mueller and Rausch, 2015). Alcohol consumers exhibit alterations in iron and iron-related proteins, including unaltered, elevated, or decreased levels of serum iron, increased duodenal iron transport proteins, downregulation of serum hepcidin, decreased or unaltered levels of transferrin, and increased or unaltered levels of transferrin saturation (Ferrao et al., 2022).

Ethanol exacerbates ALD by regulating various key molecules, leading to lipid metabolism disorders and ferroptosis in the liver. Concurrent cell death and lipid peroxidation were observed in both alcohol-treated mice and hepatocytes, which can be alleviated by a ferroptosis inhibitor (Liu CY. et al., 2020). Luo et al. demonstrated that chronic ethanol exposure not only leads to the accumulation of hepatic iron instability and lipid peroxides, but also disrupts the methionine cycle, resulting in decreased cysteine levels and GSH depletion, which triggers ferroptosis (Luo et al., 2023). Ethanol disrupts the aberrant signaling of liver sirtuin 1 (SIRT1), a nicotinamide adenine dinucleotide (NAD+, NADH)-dependent class III histone deacetylase, playing a central role in the pathogenesis of ALD (You et al., 2015). Zhou et al. observed significantly alleviated liver injury in the intestinal-specific SIRT1 knockout mice compared with the wild-type mice after ethanol feeding (Zhou et al., 2020). This effect was attributed to the reduction of hepatic ferroptosis, which was evidenced by improved iron metabolism, increased hepatic GSH levels, reduced lipid peroxidation, and the downregulation of genes associated with ferroptosis (Zhou et al., 2020). Ethanol inhibits the regulatory molecule of lipid metabolism, Lipin-1, by suppressing SIRT1 (You et al., 2015). Exacerbation of steatohepatitis in Lipin-1 overexpressing mice is associated with excessive iron accumulation, altered iron distribution, decreased levels of GSH, reduced NADPH levels, increased MDA levels, and impaired expression of genes related to ferroptosis (Zhou et al., 2019). Therefore, targeting the ferroptosis signaling pathway may offer a novel approach to treating ALD, given the significant role of ferroptosis in the disease’s development and progression.

### 4.2 Non-alcoholic fatty liver disease (NAFLD)

The liver serves as the metabolic hub for nutrients such as glucose, lipids, and amino acids. Metabolic disorders can lead to the degeneration and death of hepatocytes, resulting in various liver diseases. NAFLD is characterized by fat accumulation in more than 5% of hepatocytes in the absence of excessive alcohol consumption or other secondary causes of hepatic steatosis (e.g., obesity, hyperlipidemia, type 2 diabetes mellitus, and other metabolic syndromes) (Cusi et al., 2017). NAFLD is one of the most prevalent causes of liver disease worldwide, often paralleling the global rise in obesity (Younossi et al., 2018). It is marked by abnormal lipid deposition in the liver, which can progress from simple steatosis to nonalcoholic steatohepatitis (NASH), a precursor to cirrhosis and liver cancer (Isaacs, 2023; Tokushige et al., 2021).

NAFLD is associated with increased gluconeogenesis (Sunny et al., 2011). Insulin signaling, which is negatively regulated by protein phosphatases such as PTP1B and Shp-1, plays a critical role in this process. Liver-specific deletion of PTP1B enhances insulin signaling, thereby improving insulin’s ability to inhibit gluconeogenesis in the liver. This action helps prevent endoplasmic reticulum stress induced by a high-fat diet, with generates excessive ROS, leading to inflammation, hepatic injury, and the progression of NAFLD. Reducing ROS production has been shown to significantly decrease lipid accumulation in the liver (Bailey et al., 2015; Delibegovic et al., 2009). Systemic insulin resistance drives the development of NAFLD, creating a vicious cycle where hepatic lipid accumulation further exacerbates insulin resistance (Rui, 2014).

Ferroptosis has been identified as a trigger for initial cell death in steatohepatitis. This was demonstrated using a choline-deficient, ethionine-supplemented (CDE) diet model, where the application of a ferroptosis inhibitor showed significant effects (Tsurusaki et al., 2019). Qi et al. observed changes in the severity of NASH in mice treated with ferroptosis inducers like RSL-3 and GPX4 activators (Qi et al., 2020). The involvement of iron metabolism in NAFLD pathogenesis points to the possibility of using iron depletion strategies to manage the disease. Approximately 25%–30% of the body’s total iron is stored in the liver as ferritin. The liver plays a central role in the uptaking, utilization, and secretion of iron, making it a primary target for iron overload (Protchenko et al., 2021). Approximately one-third of patients with NAFLD exhibit signs of iron disorders, such as elevated serum ferritin with normal or slightly elevated transferrin saturation (Datz et al., 2017). Studies have indicated that serum ferritin levels can be an independent predictor of NAFLD severity (Kowdley et al., 2012; Valenti et al., 2012). Elevated ferritin is associated with metabolic insulin resistance syndrome and increased hepatic iron and fat, with patients with NASH exhibiting iron overloaded tending to have more severe disease (Nelson et al., 2011). Iron depletion has been shown to reduce insulin resistance and liver enzyme levels. Hepatic iron deposition in NAFLD is linked to specific histologic features of the liver (Nelson et al., 2011). NAFLD livers display higher intrahepatic levels of iron and ROS compared to normal livers, with excess iron promoting hepatic oxidative stress, inflammatory vesicle activation, induction of inflammatory and immune mediators, and hepatocyte injury, thereby contributing to NASH development (Handa et al., 2016; Capelletti et al., 2020).

The accumulation of lipids induces oxidative stress, a key initiator of cell death, with ferroptosis increasing the likelihood of hepatocyte swelling, inflammation, and fibrosis (Machado and Diehl, 2016). Enoyl coenzyme A hydratase 1 (ECH1) has been shown to alleviate hepatic steatosis by inhibiting ferroptosis (Liu et al., 2021). Currently, there are no particularly effective treatments for NAFLD. However, the American Association for the Study of Liver Diseases (AASLD) recommends statins for reducing the risk of cerebrovascular disease in NAFLD patients (Rinella et al., 2023). Melatonin and vitamin E have shown promise in treating NAFLD by alleviating hepatic ferroptosis and improving hepatic steatosis and inflammation, although their effectiveness in reducing hepatic fibrosis is limited (Guan et al., 2023; Lavine et al., 2011; Bril et al., 2019). A combination therapy or more targeted approaches may be necessary to address the complex pathophysiology of NAFLD. The role of ferroptosis in NAFLD opens new avenues for therapeutic intervention, targeting ferroptosis to prevent disease progression.

### 4.3 Cholestatic liver injury

Cholestasis results from impairments in the intrahepatic formation and excretion of bile, or extrahepatic obstruction of the bile ducts, leading to the retention of bile acids or bilirubin. Cholestatic liver injury is characterized by inflammation, severe obstruction of bile flow leading to altered disposal of bile acids, elevated serum transaminase levels; and ultimately, it progresses to severe fibrosis, cirrhosis, and liver failure (Woolbright and Jaeschke, 2019). For acute cholestasis-induced liver injury caused by gallstones, surgical treatment may be an option, whereas chronic conditions such as biliary atresia, primary biliary cholangitis (PBC), and primary sclerosing cholangitis (PSC) lack effective treatment options beyond ursodeoxycholic acid (UDCA). This poses a significant challenge to the demand for liver transplantation, as patients ultimately exhibit severe obstruction or even cirrhosis. Therefore, it is essential to gain a deeper understanding of the mechanisms by which cholestasis leads to liver injury to provide potential targets for disease treatment.

Hydrophobic bile acids (BAs) can induce hepatocyte death during cholestastic liver disease. Toxic BAs can directly activate hepatocyte death receptors and induce oxidative damage, leading to mitochondrial dysfunction, primarily affects the liver and extrahepatic tissues, such as the heart, skeletal muscle, and placenta. Studies have shown that bile acids can promote the generation of ROS, and reducing ROS production can protect hepatocytes from bile acid-induced cell death (Sokol et al., 2001; Sokol et al., 1998). Elevated serum BA concentrations are associated with a spectrum of cellular alterations, encompassing membrane perturbations, pro-inflammatory signaling cascades, and mitochondrial dysfunction. These effects are mediated by redox-dependent pathways, either directly or indirectly. Specific BA species, including UDCA, cholic acid (CA), deoxycholic acid (DCA), and chenodeoxycholic acid (CDCA), have been investigated for their therapeutic potential in various conditions such as cholelithiasis, PBC, and disorders of BA synthesis. Despite these preliminary investigations, further rigorous clinical trials are warranted to establish the efficacy of these BA derivatives in the treatment of the aforementioned diseases and to elucidate their mechanistic interplay with oxidative stress pathways (Orozco-Aguilar et al., 2021).

### 4.4 Autoimmune hepatitis (AIH)

The body hosts two types of immune systems: innate and adaptive. The innate immune system, primarily comprising cells like dendritic cells, macrophages, and neutrophils, is responsible for recognizing microbial infections. The adaptive immune system, on the other hand, involves T and B lymphocytes along with natural killer (NK) cells, which provide a more specific and targeted response to pathogens (Iwasaki and Medzhitov, 2015).

AIH is a condition characterized by parenchymal inflammation, driven by autoimmune responses against hepatocytes. It is marked by serum autoantibody positivity, elevated IgG levels, and sporadic necrosis with plasma cell and lymphocyte infiltration observed in liver histology (Czaja, 2014). Recent studies have increasingly highlighted the role of ferroptosis in the immune system, including its function in the immune-mediated suppression of tumors (Yang Y. et al., 2022; Stockwell and Jiang, 2019). Intracellular iron overload in T cells can lead to oxidative stress and DNA fragmentation, potentially impairing lymphocyte function (Shaw et al., 2017). Interestingly, the ferroptosis inducer erastin does not cause cell death in human peripheral blood mononuclear cells (PBMC), but instead increases lipid peroxidation, promoting PBMC proliferation and differentiation into B cells and NK cells (Wang et al., 2018). Muri et al. reported that the inhibition of ferroptosis crucial for the development, maintenance, and response of innate-like B cells and marginal zone (MZ) B cells, which require the presence of GPX4 (Muri et al., 2019). Wang et al. found that immunotherapy-activated CD8 T cells enhanced ferroptosis-specific lipid peroxidation in tumor cells, contributing to the antitumor effects of immunotherapy (Wang W. et al., 2019). Conversely, a specific subset of CD4 T cells, known as follicular helper T (TFH) cells, has also been shown to be regulated by ferroptosis, displaying lipid peroxidation and mitochondrial morphological changes similar to those characteristic of ferroptosis (Yao et al., 2021).

Studies have used animal models to investigate the role of ferroptosis in AIH. For instance, ferroptosis has been found to contribute to AIH induced by concanavalin A (Con A) in mice, with the ferroptosis inhibitor Fer-1 showing therapeutic effects (Zeng et al., 2020). The hepatic gene indoleamine 2, 3-dioxygenase 1 (IDO1) was significantly upregulated in this model, and ferroptosis elimination inhibits IDO1 upregulation and nitrification stress (Zeng et al., 2020). Caveolin-1 (Cav-1) plays a protective role against ferroptosis in AIH, with its downregulation in liver tissues associated with ferritin events and reactive nitrogen species (RNS) production, contributing to Con A-induced hepatic injury (Deng et al., 2020). Another study showed that ferroptosis induction using S100 in a mouse model led to the upregulation of COX2 and ACSL4, and the downregulation of GPX4 and FTH1, with Fer-1 restoring these expression profiles (Zhu et al., 2021). Microarray analysis in a Con A-induced AIH mouse model identified differentially expressed miRNAs and signaling pathways related to ferroptosis, which could be potential therapeutic targets (Liu Y. et al., 2020).

As our understanding of the interplay between ferroptosis and immunity grows, so does the opportunity to develop targeted interventions that could revolutionize the treatment of autoimmune diseases like AIH. In conclusion, while more research is needed to fully elucidate the mechanisms by which ferroptosis influences immune function and AIH, the current findings provide a compelling rationale for further investigation into the role of regulated cell death in immune-mediated diseases.

### 4.5 Acute liver injury

Acute liver failure is characterized by a sudden and severe injury to hepatocytes, leading to a rapid decline in liver function, which can progress to a fatal outcome within a short period. This condition can be triggered by various factors, including drugs, alcohol, ischemia/reperfusion injury (IRI), viruses, and AIH (Stravitz and Lee, 2019).

Drug-induced liver injury (DILI) is a common cause of liver damage, presenting diverse clinical manifestations such as cholestatic liver injury, acute hepatitis with or without jaundice, and nodular regenerative hyperplasia (Yuan and Kaplowitz, 2013). The molecular mechanisms underlying DILI involve mitochondrial dysfunction, increased ROS levels, elevated apoptosis and necrosis, and bile duct injury associated with immune-mediated pathways (Allison et al., 2023). Drug-induced mitochondria damage, along with their reactive metabolites, leads to oxidative stress, activation of stress signaling pathways, impaired mitochondrial function, and endoplasmic reticulum stress, which collectively cause hepatocellular death and promote subsequent inflammatory and regenerative responses (Iorga and Dara, 2019). In hepatocytes, direct toxicity and intracellular stress such as endoplasmic reticulum stress or mitochondrial toxicity can result in cell death through mechanisms like mitochondrial outer membrane permeabilization (MOMP) or mitochondrial permeability transition (MPT) (Iorga et al., 2017). Acetaminophen (APAP)-induced cell death has been shown to involve ferroptosis (Lőrincz et al., 2015). The ferroptosis inhibitor Fer-1 protects hepatocytes *in vitro* from APAP-induced damage. Although the majority of APAP is excreted after conjugation with glucuronic acid or sulfate, a small portion is metabolized by cytochrome P450 enzymes into N-acetyl-p-benzoquinone imine (NAPQI). NAPQI rapidly reacts with GSH, leading to significant GSH depletion (McGill et al., 2013; Mitchell et al., 1973). NAPQI also reacts with cellular proteins to form APAP adducts, particularly binding to mitochondrial proteins, which is recognized as a vital initiating event in cell death. Mice treated with APAP exhibit reduced GPX activity (Tirmenstein and Nelson, 1990), highlighting the role of GSH depletion and GPX inhibition in APAP-induced ferroptosis. Recent studies by Niu et al. demonstrated that injecting VDAC oligomerization inhibitor VBIT-12 and the ferroptosis inhibitor UAMC-3203 into the tail veins of APAP-injured mice significantly reduced APAP-induced ferroptosis by protecting mitochondrial function (Niu et al., 2022). These findings suggest that ferroptosis inhibitors may have clinical utility in the prevention and treatment of liver damage in various settings.

Liver IRI occurs due to reduced blood and oxygen supply to the organ, followed by reperfusion, which causes oxygen-dependent cellular damage (de Groot and Rauen, 2007), and impaired organ function. IRI can arise in various clinical scenarios, including systemic shock, heart failure, respiratory failure, trauma, and liver transplantation (Rushing and Britt, 2008; Birrer et al., 2007; Nickkholgh et al., 2008; Kupiec-Weglinski and Busuttil, 2005). The ferroptosis inhibitor Lip-1 has been shown to reduce IRI in the liver and kidney (Friedmann Angeli et al., 2014). A study identified that liver injury, lipid peroxidation, and the upregulation of the ferroptosis marker Ptgs2 in an I/R-injured mouse model were significantly prevented by Fer-1 or α-tocopherol, suggesting that ferroptosis plays a critical role in the pathogenesis of IRI (Yamada et al., 2020).

### 4.6 Viral hepatitis

Ferroptosis, a form of regulated cell death characterized by iron accumulation and lipid peroxidation, plays a crucial role in the progression of viral hepatitis. Its involvement in viral infections, including hepatitis, can manipulate ferroptosis to either boost their replication or avoid immune detection (Hu et al., 2023). In the case of hepatitis, viruses may trigger ferroptosis by disrupting iron homeostasis and increasing oxidative stress, which in turn can damage liver cells and promote viral replication and liver injury.

With hepatitis B and C, ferroptosis is associated with liver inflammation and fibrosis, where viral proteins can cause iron overload and lipid peroxidation, resulting in hepatocyte death. Heat shock protein family A member 8 (HSPA8) serves as a pivotal host factor in regulating Hepatitis B virus (HBV) replication and ferroptosis in liver cancer, suppressing ferroptosis by upregulating the expression of SLC7A11/GPX4 and reducing ROS and Fe^2+^ accumulation (Wang et al., 2023). The ferroptosis-related gene SLC1A5 correlates with the progression of HBV-related hepatocellular carcinoma (HCC), suggesting its potential as an excellent novel prognostic indicator (Su et al., 2023). Hepatitis C virus (HCV) core protein can directly induce mitochondrial injury, oxidative stress, and antioxidant gene expression (Okuda et al., 2002). Lipid peroxidation plays a critical role in determining cellular permissiveness for replication of HCV. The limitation of HCV replication through lipid peroxidation is primarily attributed to the iron-catalyzed peroxidation of hepatic very long-chain polyunsaturated fatty acids (HUFAs) that are dependent on the enzyme fatty acid desaturase 2 (FADS2) (Yamane et al., 2022). FADS2 plays a pivotal role in regulating HCV replication within infected cells by facilitating both the classical and non-classical desaturation of fatty acids and is a crucial determinant of cellular susceptibility to ferroptosis. HCV also triggers an antioxidant response via the glycogen synthase kinase (GSK) 3β-Nrf2 signaling pathway in hepatocytes, wich inhibits ferroptosis by suppressing oxidative stress (Jiang Y. et al., 2015; Huang et al., 2022).

Understanding the molecular mechanisms by which these hepatitis viruses manipulate ferroptosis offers new avenues for therapeutic intervention. The current findings suggest that targeting ferroptosis in viral hepatitis could be a promising strategy, especially by using antioxidants or ferroptosis inhibitors to protect liver cells from oxidative stress and lipid peroxidation. This could ultimately help control viral hepatitis progression.

### 4.7 Liver fibrosis

Liver fibrosis is defined by the excessive deposition of extracellular matrix proteins as a reaction to persist liver damage. When liver fibrosis progresses to cirrhosis, it presents a critical phase with severe complications such as liver failure and hepatocellular carcinoma (HCC). Activation of hepatic stellate cells (HSCs) is recognized as the principal catalyst of liver fibrogenesis (Jiao et al., 2009).

Ferroptosis plays a dual role in liver fibrosis. On one hand, it can be protective by eliminating of HSCs, and thus, ferroptosis induction by drugs like erastin, sorafenib, and artemether (ART) have been shown to alleviate hepatic fibrosis (Wang L. et al., 2019). On the other hand, ferroptosis can also contribute to liver injury and fibrosis, suggesting that it acts as a double-edged sword (Yu et al., 2020). Compounds such as ferrostatin-1 and hepatic transferrin can ameliorate liver fibrosis by inhibiting ferroptosis, implying a protective role against fibrotic development. DHODH has been identified as a crucial factor in inhibiting ferroptosis in the liver. Its inhibitor brequinar has shown promise in inhibiting and even treating liver fibrosis by inducing ferroptosis (Mao et al., 2021). Understanding the precise mechanisms by which ferroptosis contributes to or protects against liver fibrosis is crucial for developing targeted therapies.

### 4.8 Hepatocellular carcinoma (HCC)

Ferroptosis plays a critical role in the pathophysiology of cancer, and numerous strategies involving nanomaterials, clinical drugs, experimental compounds, and gene-based approaches have been developed to induce ferroptosis in cancer cells have been developed (Shen et al., 2018). In 2003, Dolma et al. identified that erastin exhibited cytotoxicity against cells expressing the mutant Ras oncogene (BjeLR) (Dolma et al., 2003). Subsequent research by Yang et al. identified small molecules RSL3 and RSL5, which triggered cell death in the presence of oncogenic RAS, exhibiting properties similar to those of erastin (Yang and Stockwell, 2008). GPX4, a key regulator of ferroptosis in cancer cells, is inactivated by erastin through the depletion of GSH (Yang et al., 2014).

Drugs like sulfasalazine, typically used for rheumatic disorders, have been repurposed to induce ferroptosis in cancer cells by inhibiting the system Xc^−^ (Bannai and Kasuga, 1985; Lo et al., 2008). Artemisinin derivatives have also shown potential in inducing ferroptosis in various tumor cell lines, with iron-related gene expression influencing the response to these derivatives (Ooko et al., 2015). Furthermore, artesunate has been shown to induce ferroptosis specifically in certain cancer types, although resistance mechanisms, such as the activation of the Nrf2-antioxidant response element (ARE) pathway in head and neck cancer cells, can reduce its effectiveness (Eling et al., 2015; Roh et al., 2017).

Non-coding RNAs (ncRNAs), including microRNAs (miRNAs), long non-coding RNAs (IncRNAs), and circular RNAs (circRNAs), have been shown to play significant roles in the regulation of ferroptosis, influencing cancer growth (Zuo et al., 2022). In lung cancer, for example, Song et al. reported that exosome miR-4443 regulates the expression of FSP1 *via* METLL3 through m6A modification, increasing cisplatin resistance and reducing the efficacy of non-small cell lung cancer (NSCLC) treatments (Song et al., 2021). Additionally, miR-27a-3p, lncRNA P53RRA, and lncRNA NEAT1 have been implicated in inducing ferroptosis and thus inhibiting lung cancer progression (Lu et al., 2021; Mao et al., 2018; Wu and Liu, 2021). In other cancers, numerous ncRNAs have also been found to play pivotal roles in cancer development and response to drug therapy by regulating ferroptosis (Yadav et al., 2021; Ni et al., 2021). These ncRNAs can target key ferroptosis-related genes such as GPX4, SLC3A2, and SLC7A11, offering novel therapeutic approaches or diagnostic biomarkers for cancer.

In 2023, liver cancer accounted for 6% of cancer-related deaths in men and 4% in women in the United States, with HCC being the most common form (Siegel et al., 2023). Higher dietary iron intake has been linked to an increased risk of HCC development (Fonseca-Nunes et al., 2014). Research suggests that activating ferroptosis can inhibit HCC cell growth, with drugs like sorafenib, a first-line HCC therapy, inducing ferroptosis in HCC cells (Lachaier et al., 2014). Resistance to sorafenib can be modulated by factors like metallothionein (MT)-1G, and recent findings implicate ferroptosis in HCC progression through various molecular axes (Sun et al., 2016). Recently, Lyu et al. reported that ferroptosis is involved in the progression of HCC through the circ0097009/miR-1261/SLC7A11 axis recently (Lyu et al., 2021). Additionally, Qi et al. found that erastin upregulates the lncRNA GABPB1-AS1, which downregulates the level of GABPB1 protein by preventing translation, thereby inhibiting cellular antioxidant capacity (Qi et al., 2019). Meanwhile, miR-214 enhances the effects of erastin and by inhibiting the expression of activating transcription factor 4 (ATF4) in HepG2 and Hep3B cancer cells (Bai et al., 2020). Overall, inducing ferroptosis represents a promising strategy for the treatment of HCC. Further research is needed to fully understand the mechanism of ferroptosis in HCC and to develop effective strategies to induce ferroptosis without causing harm to healthy cells. The potential for personalized ferroptosis-inducing therapies in cancer treatment becomes increasingly tangible.

The significance of ferroptosis in the context of hepatic pathologies is gaining substantial recognition as a key mechanism underlying ALD, NAFLD, cholestatic liver injury, AIH, acute liver injury, viral hepatitis, liver fibrosis, and HCC. This form of regulated cell death, distinguished by iron-dependent lipid peroxidation, underscores the delicate equilibrium between iron metabolism and oxidative stress within hepatocytes. It not only amplifies the severity of established liver conditions, such as alcoholic liver disease and viral hepatitis, but also presents a promising therapeutic target. By manipulating the pathways associated with ferroptosis, it may be feasible to devise innovative interventions aimed at mitigating liver damage and enhancing patient outcomes across a spectrum of hepatic disorders. Furthermore, the intricate relationship between ferroptosis and hepatic immune responses warrants further exploration, particularly in the context of chronic liver inflammation and the progression of fibrosis. Gaining this insight could facilitate the discovery of biomarkers for early diagnosis and the development of novel therapeutic strategies that harness the modulation of ferroptosis to effectively combat liver diseases.

## 5 Common ferroptosis pathways in gestational and hepatic diseases

Ferroptosis, an iron-dependent form of regulated cell death driven by lipid peroxidation, has emerged as a critical mechanism in the pathophysiology of both gestational-related diseases and liver disorder (Table 4). In gestational diseases such as PE, GDM, RPL, and ICP, ferroptosis contributes to tissue damage and oxidative stress, influencing placental function and fetal development. In liver diseases, including ALD, NAFLD, cholestatic liver diseases, AIH, viral hepatitis, liver fibrosis, cirrhosis, and HCC, ferroptosis similarly drives hepatocyte death.

**TABLE 4:** Common ferroptosis pathways and potential therapeutic targets in gestational and hepatic diseases.

Pathway/Target	Role in gestational diseases	Role in liver diseases	Potential therapeutic target
Iron metabolism (Hepcidin/Ferroportin)	Dysregulated iron metabolism leads to oxidative stress in PE, GDM, ICP	Iron overload promotes ferroptosis in ALD, NAFLD, HCC, and viral hepatitis	Iron chelators (e.g., deferoxamine) to reduce iron overload
GPX4	Reduced GPX4 activity contributes to oxidative stress in PE and ICP	Reduced GPX4 activity leads to lipid peroxidation in NAFLD, cirrhosis, and HCC	GPX4 activators (e.g., liproxstatins) to prevent ferroptosis
Lipid peroxidation (ACSL4)	Drives oxidative damage in ICP and contributes to PRL	Promotes lipid peroxidation and ferroptosis in liver fibrosis, cirrhosis, HCC	ACSL4 inhibitors (e.g., Fer-1) to block lipid peroxidation
SLC7A11/System Xc^-^	Impaired in RPL, leading to placental cell death	Reduced activity contributes to ferroptosis in HCC and liver fibrosis	Enhancing SLC7A11 function or inhibiting in HCC for selective cancer targeting
Nrf2 pathway	Nrf2 protects against oxidative stress in GDM, PE	Nrf2 activation mitigates oxidative damage in NAFLD, ALD, cirrhosis	Nrf2 inducers (e.g., dimethyl fumarate) to boost antioxidant defenses
FSP1	Inhibits ferroptosis in PE, preventing placental oxidative damage	FSP1 protects against lipid peroxidation in NAFLD and HCC	FSP1 activators to reduce ferroptosis and oxidative damage
GSH	Low GSH levels exacerbate oxidative stress in PE, GDM, ICP	Reduced GSH levels promote ferroptosis in liver diseases like HCC	GSH supplementation or N-acetylcysteine to increase antioxidant capacity
HO-1	Protective role in mitigating oxidative stress in PE, RPL	Regulates oxidative stress and iron metabolism in viral hepatitis and HCC	HO-1 inducers could protect against ferroptosis and liver damage

## 6 Conclusion

Ferroptosis, characterized by disruptions in lipid metabolism, iron metabolism, and the antioxidant system, has gained attention as a promising therapeutic target due to its emerging role in various diseases, including placental dysfunction and liver disorders. While increasing evidence highlights its significance, the molecular mechanisms driving ferroptosis in both gestational physiology and liver diseases remain poorly understood, leaving critical questions unanswered. Initially discovered in the context of cancer and neurodegenerative diseases, ferroptosis has since been implicated in pregnancy complications and liver pathologies. However, the precise balance between ferroptosis deficiency and excess in these these conditions is still unclear. Further studies are necessary to elucidate the precise role of ferroptosis in pregnancy-related disorders and liver diseases, to better understand its mechanisms and therapeautic potential.

## References

[B1] Allison R. Guraka A. Shawa I. T. Tripathi G. Moritz W. Kermanizadeh A. (2023). Drug induced liver injury - a 2023 update. J. Toxicol. Environ. Health B Crit. Rev. 26 (8), 442–467. 10.1080/10937404.2023.2261848 37786264

[B2] Alp N. J. Mussa S. Khoo J. Cai S. Guzik T. Jefferson A. (2003). Tetrahydrobiopterin-dependent preservation of nitric oxide-mediated endothelial function in diabetes by targeted transgenic GTP-cyclohydrolase I overexpression. J. Clin. Invest. 112 (5), 725–735. 10.1172/JCI17786 12952921 PMC182196

[B3] Anandhan A. Dodson M. Shakya A. Chen J. Liu P. Wei Y. (2023). NRF2 controls iron homeostasis and ferroptosis through HERC2 and VAMP8. Sci. Adv. 9 (5), eade9585. 10.1126/sciadv.ade9585 36724221 PMC9891695

[B4] Ataide R. Fielding K. Pasricha S. R. Bennett C. (2023). Iron deficiency, pregnancy, and neonatal development. Int. J. Gynaecol. Obstet. 162 (Suppl. 2), 14–22. 10.1002/ijgo.14944 37538017

[B5] Badgley M. A. Kremer D. M. Maurer H. C. DelGiorno K. E. Lee H. J. Purohit V. (2020). Cysteine depletion induces pancreatic tumor ferroptosis in mice. Science 368 (6486), 85–89. 10.1126/science.aaw9872 32241947 PMC7681911

[B6] Bai T. Liang R. Zhu R. Wang W. Zhou L. Sun Y. (2020). MicroRNA-214-3p enhances erastin-induced ferroptosis by targeting ATF4 in hepatoma cells. J. Cell Physiol. 235 (7-8), 5637–5648. 10.1002/jcp.29496 31960438

[B7] Bailey A. P. Koster G. Guillermier C. Hirst E. M. MacRae J. I. Lechene C. P. (2015). Antioxidant role for lipid droplets in a stem cell niche of Drosophila. Cell 163 (2), 340–353. 10.1016/j.cell.2015.09.020 26451484 PMC4601084

[B8] Bannai S. (1986). Exchange of cystine and glutamate across plasma membrane of human fibroblasts. J. Biol. Chem. 261 (5), 2256–2263. 10.1016/s0021-9258(17)35926-4 2868011

[B9] Bannai S. Kasuga H. (1985). Anti-inflammatory drug inhibition of transport of cystine and glutamate in cultured human fibroblasts. Biochem. Pharmacol. 34 (10), 1852–1854. 10.1016/0006-2952(85)90663-x 2860907

[B10] Beharier O. Tyurin V. A. Goff J. P. Guerrero-Santoro J. Kajiwara K. Chu T. (2020). PLA2G6 guards placental trophoblasts against ferroptotic injury. Proc. Natl. Acad. Sci. U. S. A. 117 (44), 27319–27328. 10.1073/pnas.2009201117 33087576 PMC7959495

[B11] Bentinger M. Brismar K. Dallner G. (2007). The antioxidant role of coenzyme Q. Mitochondrion 7 (Suppl. l), S41–S50. 10.1016/j.mito.2007.02.006 17482888

[B12] Bersuker K. Hendricks J. M. Li Z. Magtanong L. Ford B. Tang P. H. (2019). The CoQ oxidoreductase FSP1 acts parallel to GPX4 to inhibit ferroptosis. Nature 575 (7784), 688–692. 10.1038/s41586-019-1705-2 31634900 PMC6883167

[B13] Birrer R. Takuda Y. Takara T. (2007). Hypoxic hepatopathy: pathophysiology and prognosis. Intern Med. 46 (14), 1063–1070. 10.2169/internalmedicine.46.0059 17634701

[B14] Bril F. Biernacki D. M. Kalavalapalli S. Lomonaco R. Subbarayan S. K. Lai J. (2019). Role of vitamin E for nonalcoholic steatohepatitis in patients with type 2 diabetes: a randomized controlled trial. Diabetes Care 42 (8), 1481–1488. 10.2337/dc19-0167 31332029

[B15] Brown C. W. Amante J. J. Chhoy P. Elaimy A. L. Liu H. Zhu L. J. (2019). Prominin2 drives ferroptosis resistance by stimulating iron export. Dev. Cell 51 (5), 575–586. 10.1016/j.devcel.2019.10.007 31735663 PMC8316835

[B16] Brown S. H. Eather S. R. Freeman D. J. Meyer B. J. Mitchell T. W. (2016). A lipidomic analysis of placenta in preeclampsia: evidence for lipid storage. PLoS One 11 (9), e0163972. 10.1371/journal.pone.0163972 27685997 PMC5042456

[B17] Burton G. J. Jauniaux E. Watson A. L. (1999). Maternal arterial connections to the placental intervillous space during the first trimester of human pregnancy: the Boyd collection revisited. Am. J. Obstet. Gynecol. 181 (3), 718–724. 10.1016/s0002-9378(99)70518-1 10486489

[B18] Calkin A. C. Tontonoz P. (2012). Transcriptional integration of metabolism by the nuclear sterol-activated receptors LXR and FXR. Nat. Rev. Mol. Cell Biol. 13 (4), 213–224. 10.1038/nrm3312 22414897 PMC3597092

[B19] Cao C. O'Brien K. O. (2013). Pregnancy and iron homeostasis: an update. Nutr. Rev. 71 (1), 35–51. 10.1111/j.1753-4887.2012.00550.x 23282250

[B20] Capelletti M. M. Manceau H. Puy H. Peoc’h K. (2020). Ferroptosis in liver diseases: an overview. Int. J. Mol. Sci. 21 (14), 4908. 10.3390/ijms21144908 32664576 PMC7404091

[B21] Chang L. C. Chiang S. K. Chen S. E. Yu Y. L. Chou R. H. Chang W. C. (2018). Heme oxygenase-1 mediates BAY 11-7085 induced ferroptosis. Cancer Lett. 416, 124–137. 10.1016/j.canlet.2017.12.025 29274359

[B22] Chang S. C. O'Brien K. O. Nathanson M. S. Mancini J. Witter F. R. (2003). Hemoglobin concentrations influence birth outcomes in pregnant African-American adolescents. J. Nutr. 133 (7), 2348–2355. 10.1093/jn/133.7.2348 12840205

[B23] Chen A. Zhao H. Wang J. Zhang R. Liu J. Zhao X. (2020). Haplotype analysis of candidate genes involved in inflammation and oxidative stress and the susceptibility to preeclampsia. J. Immunol. Res. 2020, 4683798. 10.1155/2020/4683798 32185238 PMC7061132

[B24] Chong W. S. Kwan P. C. Chan L. Y. Chiu P. Y. Cheung T. K. Lau T. K. (2005). Expression of divalent metal transporter 1 (DMT1) isoforms in first trimester human placenta and embryonic tissues. Hum. Reprod. 20 (12), 3532–3538. 10.1093/humrep/dei246 16123094

[B25] Chorley B. N. Campbell M. R. Wang X. Karaca M. Sambandan D. Bangura F. (2012). Identification of novel NRF2-regulated genes by ChIP-Seq: influence on retinoid X receptor alpha. Nucleic Acids Res. 40 (15), 7416–7429. 10.1093/nar/gks409 22581777 PMC3424561

[B26] Chu B. Kon N. Chen D. Li T. Liu T. Jiang L. (2019). ALOX12 is required for p53-mediated tumour suppression through a distinct ferroptosis pathway. Nat. Cell Biol. 21 (5), 579–591. 10.1038/s41556-019-0305-6 30962574 PMC6624840

[B27] Conrad M. Pratt D. A. (2019). The chemical basis of ferroptosis. Nat. Chem. Biol. 15 (12), 1137–1147. 10.1038/s41589-019-0408-1 31740834

[B28] Cronin S. J. F. Seehus C. Weidinger A. Talbot S. Reissig S. Seifert M. (2018). The metabolite BH4 controls T cell proliferation in autoimmunity and cancer. Nature 563 (7732), 564–568. 10.1038/s41586-018-0701-2 30405245 PMC6438708

[B29] Cui S. Simmons G. Jr. Vale G. Deng Y. Kim J. Kim H. (2022). FAF1 blocks ferroptosis by inhibiting peroxidation of polyunsaturated fatty acids. Proc. Natl. Acad. Sci. U. S. A. 119 (17), e2107189119. 10.1073/pnas.2107189119 35467977 PMC9169925

[B30] Cusi K. Sanyal A. J. Zhang S. Hartman M. L. Bue-Valleskey J. M. Hoogwerf B. J. (2017). Non-alcoholic fatty liver disease (NAFLD) prevalence and its metabolic associations in patients with type 1 diabetes and type 2 diabetes. Diabetes Obes. Metab. 19 (11), 1630–1634. 10.1111/dom.12973 28417532

[B31] Czaja A. J. (2014). Targeting apoptosis in autoimmune hepatitis. Dig. Dis. Sci. 59 (12), 2890–2904. 10.1007/s10620-014-3284-2 25038736

[B32] Dai E. Zhang W. Cong D. Kang R. Wang J. Tang D. (2020). AIFM2 blocks ferroptosis independent of ubiquinol metabolism. Biochem. Biophys. Res. Commun. 523 (4), 966–971. 10.1016/j.bbrc.2020.01.066 31964528

[B33] Daru J. Zamora J. Fernández-Félix B. M. Vogel J. Oladapo O. T. Morisaki N. (2018). Risk of maternal mortality in women with severe anaemia during pregnancy and post partum: a multilevel analysis. Lancet Glob. Health 6 (5), e548–e554. 10.1016/S2214-109X(18)30078-0 29571592

[B34] Datz C. Müller E. Aigner E. (2017). Iron overload and non-alcoholic fatty liver disease. Minerva Endocrinol. 42 (2), 173–183. 10.23736/S0391-1977.16.02565-7 27834478

[B35] de Groot H. Rauen U. (2007). Ischemia-reperfusion injury: processes in pathogenetic networks: a review. Transpl. Proc. 39 (2), 481–484. 10.1016/j.transproceed.2006.12.012 17362763

[B36] Delibegovic M. Zimmer D. Kauffman C. Rak K. Hong E. G. Cho Y. R. (2009). Liver-specific deletion of protein-tyrosine phosphatase 1B (PTP1B) improves metabolic syndrome and attenuates diet-induced endoplasmic reticulum stress. Diabetes 58 (3), 590–599. 10.2337/db08-0913 19074988 PMC2646057

[B37] Deng G. Li Y. Ma S. Gao Z. Zeng T. Chen L. (2020). Caveolin-1 dictates ferroptosis in the execution of acute immune-mediated hepatic damage by attenuating nitrogen stress. Free Radic. Biol. Med. 148, 151–161. 10.1016/j.freeradbiomed.2019.12.026 31877357

[B38] Dewey K. G. Oaks B. M. (2017). U-shaped curve for risk associated with maternal hemoglobin, iron status, or iron supplementation. Am. J. Clin. Nutr. 106 (Suppl. 6), 1694S–1702S. 10.3945/ajcn.117.156075 29070565 PMC5701708

[B39] Dixon S. J. Lemberg K. M. Lamprecht M. R. Skouta R. Zaitsev E. M. Gleason C. E. (2012). Ferroptosis: an iron-dependent form of nonapoptotic cell death. Cell 149 (5), 1060–1072. 10.1016/j.cell.2012.03.042 22632970 PMC3367386

[B40] Doll S. Freitas F. P. Shah R. Aldrovandi M. da Silva M. C. Ingold I. (2019). FSP1 is a glutathione-independent ferroptosis suppressor. Nature 575 (7784), 693–698. 10.1038/s41586-019-1707-0 31634899

[B41] Doll S. Proneth B. Tyurina Y. Y. Panzilius E. Kobayashi S. Ingold I. (2017). ACSL4 dictates ferroptosis sensitivity by shaping cellular lipid composition. Nat. Chem. Biol. 13 (1), 91–98. 10.1038/nchembio.2239 27842070 PMC5610546

[B42] Dolma S. Lessnick S. L. Hahn W. C. Stockwell B. R. (2003). Identification of genotype-selective antitumor agents using synthetic lethal chemical screening in engineered human tumor cells. Cancer Cell 3 (3), 285–296. 10.1016/s1535-6108(03)00050-3 12676586

[B43] Donovan A. Brownlie A. Zhou Y. Shepard J. Pratt S. J. Moynihan J. (2000). Positional cloning of zebrafish ferroportin1 identifies a conserved vertebrate iron exporter. Nature 403 (6771), 776–781. 10.1038/35001596 10693807

[B44] Donovan A. Lima C. A. Pinkus J. L. Pinkus G. S. Zon L. I. Robine S. (2005). The iron exporter ferroportin/Slc40a1 is essential for iron homeostasis. Cell Metab. 1 (3), 191–200. 10.1016/j.cmet.2005.01.003 16054062

[B45] Du L. Deng W. Zeng S. Xu P. Huang L. Liang Y. (2021). Single-cell transcriptome analysis reveals defective decidua stromal niche attributes to recurrent spontaneous abortion. Cell Prolif. 54 (11), e13125. 10.1111/cpr.13125 34546587 PMC8560595

[B46] Eling N. Reuter L. Hazin J. Hamacher-Brady A. Brady N. R. (2015). Identification of artesunate as a specific activator of ferroptosis in pancreatic cancer cells. Oncoscience 2 (5), 517–532. 10.18632/oncoscience.160 26097885 PMC4468338

[B47] Evans P. Cindrova-Davies T. Muttukrishna S. Burton G. J. Porter J. Jauniaux E. (2011). Hepcidin and iron species distribution inside the first-trimester human gestational sac. Mol. Hum. Reprod. 17 (4), 227–232. 10.1093/molehr/gaq101 21177636 PMC3050573

[B48] Fang Y. Fang D. (2022). Comprehensive analysis of placental gene-expression profiles and identification of EGFR-mediated autophagy and ferroptosis suppression in intrahepatic cholestasis of pregnancy. Gene 834, 146594. 10.1016/j.gene.2022.146594 35643225

[B49] Ferrao K. Ali N. Mehta K. J. (2022). Iron and iron-related proteins in alcohol consumers: cellular and clinical aspects. J. Mol. Med. Berl. 100 (12), 1673–1689. 10.1007/s00109-022-02254-8 36214835 PMC9691479

[B50] Fisher A. L. Nemeth E. (2017). Iron homeostasis during pregnancy. Am. J. Clin. Nutr. 106 (Suppl. 6), 1567S–1574S. 10.3945/ajcn.117.155812 29070542 PMC5701706

[B51] Fonseca-Nunes A. Jakszyn P. Agudo A. (2014). Iron and cancer risk--a systematic review and meta-analysis of the epidemiological evidence. Cancer Epidemiol. Biomarkers Prev. 23 (1), 12–31. 10.1158/1055-9965.EPI-13-0733 24243555

[B52] Forcina G. C. Dixon S. J. (2019). GPX4 at the crossroads of lipid homeostasis and ferroptosis. Proteomics 19 (18), e1800311. 10.1002/pmic.201800311 30888116

[B53] Frazer D. M. Anderson G. J. (2014). The regulation of iron transport. Biofactors 40 (2), 206–214. 10.1002/biof.1148 24132807

[B54] Friedmann Angeli J. P. Conrad M. (2018). Selenium and GPX4, a vital symbiosis. Free Radic. Biol. Med. 127, 153–159. 10.1016/j.freeradbiomed.2018.03.001 29522794

[B55] Friedmann Angeli J. P. Schneider M. Proneth B. Tyurina Y. Y. Tyurin V. A. Hammond V. J. (2014). Inactivation of the ferroptosis regulator Gpx4 triggers acute renal failure in mice. Nat. Cell Biol. 16 (12), 1180–1191. 10.1038/ncb3064 25402683 PMC4894846

[B56] Gan B. (2022). ACSL4, PUFA, and ferroptosis: new arsenal in anti-tumor immunity. Signal Transduct. Target Ther. 7 (1), 128. 10.1038/s41392-022-01004-z 35459217 PMC9033814

[B57] Geenes V. Williamson C. (2009). Intrahepatic cholestasis of pregnancy. World J. Gastroenterol. 15 (17), 2049–2066. 10.3748/wjg.15.2049 19418576 PMC2678574

[B58] Georgieff M. K. Krebs N. F. Cusick S. E. (2019). The benefits and risks of iron supplementation in pregnancy and childhood. Annu. Rev. Nutr. 39, 121–146. 10.1146/annurev-nutr-082018-124213 31091416 PMC7173188

[B59] Girling J. Knight C. L. Chappell L. Royal College of Obstetricians and Gynaecologists (2022). Intrahepatic cholestasis of pregnancy: green-top guideline No. 43 june 2022. Bjog 129 (13), e95–e114. 10.1111/1471-0528.17206 35942656

[B60] Goodwin B. Jones S. A. Price R. R. Watson M. A. McKee D. D. Moore L. B. (2000). A regulatory cascade of the nuclear receptors FXR, SHP-1, and LRH-1 represses bile acid biosynthesis. Mol. Cell 6 (3), 517–526. 10.1016/s1097-2765(00)00051-4 11030332

[B61] Guan Q. Wang Z. Hu K. Cao J. Dong Y. Chen Y. (2023). Melatonin ameliorates hepatic ferroptosis in NAFLD by inhibiting ER stress via the MT2/cAMP/PKA/IRE1 signaling pathway. Int. J. Biol. Sci. 19 (12), 3937–3950. 10.7150/ijbs.85883 37564204 PMC10411470

[B62] Gumilar K. E. Priangga B. Lu C. H. Dachlan E. G. Tan M. (2023). Iron metabolism and ferroptosis: a pathway for understanding preeclampsia. Biomed. Pharmacother. 167, 115565. 10.1016/j.biopha.2023.115565 37751641

[B63] Gutierrez-Aguirre C. H. García-Lozano J. A. Treviño-Montemayor O. R. Iglesias-Benavides J. L. Cantú-Rodríguez O. G. González-Llano O. (2017). Comparative analysis of iron status and other hematological parameters in preeclampsia. Hematology. 22 (1), 36–40. 10.1080/10245332.2016.1220120 27558940

[B64] Haeggström J. Z. Funk C. D. (2011). Lipoxygenase and leukotriene pathways: biochemistry, biology, and roles in disease. Chem. Rev. 111 (10), 5866–5898. 10.1021/cr200246d 21936577

[B65] Handa P. Morgan-Stevenson V. Maliken B. D. Nelson J. E. Washington S. Westerman M. (2016). Iron overload results in hepatic oxidative stress, immune cell activation, and hepatocellular ballooning injury, leading to nonalcoholic steatohepatitis in genetically obese mice. Am. J. Physiol. Gastrointest. Liver Physiol. 310 (2), G117–G127. 10.1152/ajpgi.00246.2015 26564716

[B66] Hanieh S. Ha T. T. Simpson J. A. Casey G. J. Khuong N. C. Thoang D. D. (2013). The effect of intermittent antenatal iron supplementation on maternal and infant outcomes in rural Viet Nam: a cluster randomised trial. PLoS Med. 10 (6), e1001470. 10.1371/journal.pmed.1001470 23853552 PMC3708703

[B67] Harvey C. J. Thimmulappa R. K. Singh A. Blake D. J. Ling G. Wakabayashi N. (2009). Nrf2-regulated glutathione recycling independent of biosynthesis is critical for cell survival during oxidative stress. Free Radic. Biol. Med. 46 (4), 443–453. 10.1016/j.freeradbiomed.2008.10.040 19028565 PMC2634824

[B68] Hempstock J. Jauniaux E. Greenwold N. Burton G. J. (2003). The contribution of placental oxidative stress to early pregnancy failure. Hum. Pathol. 34 (12), 1265–1275. 10.1016/j.humpath.2003.08.006 14691912

[B69] Hishikawa D. Shindou H. Kobayashi S. Nakanishi H. Taguchi R. Shimizu T. (2008). Discovery of a lysophospholipid acyltransferase family essential for membrane asymmetry and diversity. Proc. Natl. Acad. Sci. U. S. A. 105 (8), 2830–2835. 10.1073/pnas.0712245105 18287005 PMC2268545

[B70] Holland O. J. Cuffe J. S. M. Dekker Nitert M. Callaway L. Kwan Cheung K. A. Radenkovic F. (2018). Placental mitochondrial adaptations in preeclampsia associated with progression to term delivery. Cell Death Dis. 9 (12), 1150. 10.1038/s41419-018-1190-9 30455461 PMC6242930

[B71] Horikoshi N. Cong J. Kley N. Shenk T. (1999). Isolation of differentially expressed cDNAs from p53-dependent apoptotic cells: activation of the human homologue of the Drosophila peroxidasin gene. Biochem. Biophys. Res. Commun. 261 (3), 864–869. 10.1006/bbrc.1999.1123 10441517

[B72] Hou W. Xie Y. Song X. Sun X. Lotze M. T. Zeh H. J. 3rd (2016). Autophagy promotes ferroptosis by degradation of ferritin. Autophagy 12 (8), 1425–1428. 10.1080/15548627.2016.1187366 27245739 PMC4968231

[B73] Hu Y. He B. Cao Q. Li Y. Tang Y. Cao T. (2023). Crosstalk of ferroptosis and oxidative stress in infectious diseases. Front. Mol. Biosci. 10, 1315935. 10.3389/fmolb.2023.1315935 38131014 PMC10733455

[B74] Hu Y. Y. Liu J. C. Xing A. Y. (2015). Oxidative stress markers in intrahepatic cholestasis of pregnancy: a prospective controlled study. Eur. Rev. Med. Pharmacol. Sci. 19 (17), 3181–3186. 26400520

[B75] Huang S. Wang Y. Xie S. Lai Y. Mo C. Zeng T. (2022). Hepatic TGFβr1 deficiency attenuates lipopolysaccharide/D-galactosamine-induced acute liver failure through inhibiting gsk3β-nrf2-mediated hepatocyte apoptosis and ferroptosis. Cell Mol. Gastroenterol. Hepatol. 13 (6), 1649–1672. 10.1016/j.jcmgh.2022.02.009 35202887 PMC9046809

[B76] Ingold I. Berndt C. Schmitt S. Doll S. Poschmann G. Buday K. (2018). Selenium utilization by GPX4 is required to prevent hydroperoxide-induced ferroptosis. Cell 172 (3), 409–422. 10.1016/j.cell.2017.11.048 29290465

[B77] Iorga A. Dara L. (2019). Cell death in drug-induced liver injury. Adv. Pharmacol. 85, 31–74. 10.1016/bs.apha.2019.01.006 31307591

[B78] Iorga A. Dara L. Kaplowitz N. (2017). Drug-induced liver injury: cascade of events leading to cell death, apoptosis or necrosis. Int. J. Mol. Sci. 18 (5), 1018. 10.3390/ijms18051018 28486401 PMC5454931

[B79] Isaacs S. (2023). Nonalcoholic fatty liver disease. Endocrinol. Metab. Clin. North Am. 52 (1), 149–164. 10.1016/j.ecl.2022.06.007 36754491

[B80] Iwasaki A. Medzhitov R. (2015). Control of adaptive immunity by the innate immune system. Nat. Immunol. 16 (4), 343–353. 10.1038/ni.3123 25789684 PMC4507498

[B81] Jaeggi T. Kortman G. A. Moretti D. Chassard C. Holding P. Dostal A. (2015). Iron fortification adversely affects the gut microbiome, increases pathogen abundance and induces intestinal inflammation in Kenyan infants. Gut 64 (5), 731–742. 10.1136/gutjnl-2014-307720 25143342

[B82] Jiang L. Kon N. Li T. Wang S. J. Su T. Hibshoosh H. (2015a). Ferroptosis as a p53-mediated activity during tumour suppression. Nature 520 (7545), 57–62. 10.1038/nature14344 25799988 PMC4455927

[B83] Jiang Y. Bao H. Ge Y. Tang W. Cheng D. Luo K. (2015b). Therapeutic targeting of GSK3β enhances the Nrf2 antioxidant response and confers hepatic cytoprotection in hepatitis C. Gut 64 (1), 168–179. 10.1136/gutjnl-2013-306043 24811996 PMC4263291

[B84] Jiao J. Friedman S. L. Aloman C. (2009). Hepatic fibrosis. Curr. Opin. Gastroenterol. 25 (3), 223–229. 10.1097/mog.0b013e3283279668 19396960 PMC2883289

[B85] Kagan V. E. Mao G. Qu F. Angeli J. P. Doll S. Croix C. S. (2017). Oxidized arachidonic and adrenic PEs navigate cells to ferroptosis. Nat. Chem. Biol. 13 (1), 81–90. 10.1038/nchembio.2238 27842066 PMC5506843

[B86] Kaymak I. Maier C. R. Schmitz W. Campbell A. D. Dankworth B. Ade C. P. (2020). Mevalonate pathway provides ubiquinone to maintain pyrimidine synthesis and survival in p53-deficient cancer cells exposed to metabolic stress. Cancer Res. 80 (2), 189–203. 10.1158/0008-5472.CAN-19-0650 31744820

[B87] Kolb J. P. Oguin T. H. 3rd Oberst A. Martinez J. (2017). Programmed cell death and inflammation: winter is coming. Trends Immunol. 38 (10), 705–718. 10.1016/j.it.2017.06.009 28734635 PMC5710799

[B88] Kowdley K. V. Belt P. Wilson L. A. Yeh M. M. Neuschwander-Tetri B. A. Chalasani N. (2012). Serum ferritin is an independent predictor of histologic severity and advanced fibrosis in patients with nonalcoholic fatty liver disease. Hepatology 55 (1), 77–85. 10.1002/hep.24706 21953442 PMC3245347

[B89] Kraft V. A. N. Bezjian C. T. Pfeiffer S. Ringelstetter L. Müller C. Zandkarimi F. (2020). GTP cyclohydrolase 1/tetrahydrobiopterin counteract ferroptosis through lipid remodeling. ACS Cent. Sci. 6 (1), 41–53. 10.1021/acscentsci.9b01063 31989025 PMC6978838

[B90] Kupiec-Weglinski J. W. Busuttil R. W. (2005). Ischemia and reperfusion injury in liver transplantation. Transpl. Proc. 37 (4), 1653–1656. 10.1016/j.transproceed.2005.03.134 15919422

[B91] Lachaier E. Louandre C. Godin C. Saidak Z. Baert M. Diouf M. (2014). Sorafenib induces ferroptosis in human cancer cell lines originating from different solid tumors. Anticancer Res. 34 (11), 6417–6422. 25368241

[B92] Lao T. T. Chan P. L. Tam K. F. (2001). Gestational diabetes mellitus in the last trimester - a feature of maternal iron excess? Diabet. Med. 18 (3), 218–223. 10.1046/j.1464-5491.2001.00453.x 11318843

[B93] Lavine J. E. Schwimmer J. B. Van Natta M. L. Molleston J. P. Murray K. F. Rosenthal P. (2011). Effect of vitamin E or metformin for treatment of nonalcoholic fatty liver disease in children and adolescents: the TONIC randomized controlled trial. Jama 305 (16), 1659–1668. 10.1001/jama.2011.520 21521847 PMC3110082

[B94] Levy J. E. Jin O. Fujiwara Y. Kuo F. Andrews N. C. (1999). Transferrin receptor is necessary for development of erythrocytes and the nervous system. Nat. Genet. 21 (4), 396–399. 10.1038/7727 10192390

[B95] Lewerenz J. Hewett S. J. Huang Y. Lambros M. Gout P. W. Kalivas P. W. (2013). The cystine/glutamate antiporter system x(c)(-) in health and disease: from molecular mechanisms to novel therapeutic opportunities. Antioxid. Redox Signal 18 (5), 522–555. 10.1089/ars.2011.4391 22667998 PMC3545354

[B96] Li W. Liang L. Liu S. Yi H. Zhou Y. (2023). FSP1: a key regulator of ferroptosis. Trends Mol. Med. 29 (9), 753–764. 10.1016/j.molmed.2023.05.013 37357101

[B97] Li Y. Feng D. Wang Z. Zhao Y. Sun R. Tian D. (2019). Ischemia-induced ACSL4 activation contributes to ferroptosis-mediated tissue injury in intestinal ischemia/reperfusion. Cell Death Differ. 26 (11), 2284–2299. 10.1038/s41418-019-0299-4 30737476 PMC6889315

[B98] Liang H. Yoo S. E. Na R. Walter C. A. Richardson A. Ran Q. (2009). Short form glutathione peroxidase 4 is the essential isoform required for survival and somatic mitochondrial functions. J. Biol. Chem. 284 (45), 30836–30844. 10.1074/jbc.M109.032839 19744930 PMC2781482

[B99] Liu B. Yi W. Mao X. Yang L. Rao C. (2021). Enoyl coenzyme A hydratase 1 alleviates nonalcoholic steatohepatitis in mice by suppressing hepatic ferroptosis. Am. J. Physiol. Endocrinol. Metab. 320 (5), E925–E937. 10.1152/ajpendo.00614.2020 33813878

[B100] Liu C. Y. Wang M. Yu H. M. Han F. X. Wu Q. S. Cai X. J. (2020a). Ferroptosis is involved in alcohol-induced cell death *in vivo* and *in vitro* . Biosci. Biotechnol. Biochem. 84 (8), 1621–1628. 10.1080/09168451.2020.1763155 32419644

[B101] Liu Y. Chen H. Hao J. Li Z. Hou T. Hao H. (2020b). Characterization and functional prediction of the microRNAs differentially expressed in a mouse model of concanavalin A-induced autoimmune hepatitis. Int. J. Med. Sci. 17 (15), 2312–2327. 10.7150/ijms.47766 32922197 PMC7484648

[B102] Liuzzi J. P. Aydemir F. Nam H. Knutson M. D. Cousins R. J. (2006). Zip14 (Slc39a14) mediates non-transferrin-bound iron uptake into cells. Proc. Natl. Acad. Sci. U. S. A. 103 (37), 13612–13617. 10.1073/pnas.0606424103 16950869 PMC1564235

[B103] Lo M. Wang Y. Z. Gout P. W. (2008). The x(c)- cystine/glutamate antiporter: a potential target for therapy of cancer and other diseases. J. Cell Physiol. 215 (3), 593–602. 10.1002/jcp.21366 18181196

[B104] Lőrincz T. Jemnitz K. Kardon T. Mandl J. Szarka A. (2015). Ferroptosis is involved in acetaminophen induced cell death. Pathol. Oncol. Res. 21 (4), 1115–1121. 10.1007/s12253-015-9946-3 25962350

[B105] Lu S. C. (2009). Regulation of glutathione synthesis. Mol. Asp. Med. 30 (1-2), 42–59. 10.1016/j.mam.2008.05.005 PMC2704241 18601945

[B106] Lu X. Kang N. Ling X. Pan M. Du W. Gao S. (2021). MiR-27a-3p promotes non-small cell lung cancer through slc7a11-mediated-ferroptosis. Front. Oncol. 11, 759346. 10.3389/fonc.2021.759346 34722314 PMC8548660

[B107] Luedde T. Kaplowitz N. Schwabe R. F. (2014). Cell death and cell death responses in liver disease: mechanisms and clinical relevance. Gastroenterology 147 (4), 765–783. 10.1053/j.gastro.2014.07.018 25046161 PMC4531834

[B108] Luo J. Song G. Chen N. Xie M. Niu X. Zhou S. (2023). Ferroptosis contributes to ethanol-induced hepatic cell death via labile iron accumulation and GPx4 inactivation. Cell Death Discov. 9 (1), 311. 10.1038/s41420-023-01608-6 37626043 PMC10457354

[B109] Lyu N. Zeng Y. Kong Y. Chen Q. Deng H. Ou S. (2021). Ferroptosis is involved in the progression of hepatocellular carcinoma through the circ0097009/miR-1261/SLC7A11 axis. Ann. Transl. Med. 9 (8), 675. 10.21037/atm-21-997 33987373 PMC8106082

[B110] Machado M. V. Diehl A. M. (2016). Pathogenesis of nonalcoholic steatohepatitis. Gastroenterology 150 (8), 1769–1777. 10.1053/j.gastro.2016.02.066 26928243 PMC4887389

[B111] Magtanong L. Ko P. J. To M. Cao J. Y. Forcina G. C. Tarangelo A. (2019). Exogenous monounsaturated fatty acids promote a ferroptosis-resistant cell state. Cell Chem. Biol. 26 (3), 420–432. 10.1016/j.chembiol.2018.11.016 30686757 PMC6430697

[B112] Makishima M. Okamoto A. Y. Repa J. J. Tu H. Learned R. M. Luk A. (1999). Identification of a nuclear receptor for bile acids. Science 284 (5418), 1362–1365. 10.1126/science.284.5418.1362 10334992

[B113] Manivarma T. Kapralov A. A. Samovich S. N. Tyurina Y. Y. Tyurin V. A. VanDemark A. P. (2023). Membrane regulation of 15LOX-1/PEBP1 complex prompts the generation of ferroptotic signals, oxygenated PEs. Free Radic. Biol. Med. 208, 458–467. 10.1016/j.freeradbiomed.2023.09.001 37678654 PMC10952060

[B114] Mao C. Liu X. Zhang Y. Lei G. Yan Y. Lee H. (2021). DHODH-mediated ferroptosis defence is a targetable vulnerability in cancer. Nature 593 (7860), 586–590. 10.1038/s41586-021-03539-7 33981038 PMC8895686

[B115] Mao C. Wang X. Liu Y. Wang M. Yan B. Jiang Y. (2018). A G3BP1-interacting lncRNA promotes ferroptosis and apoptosis in cancer via nuclear sequestration of p53. Cancer Res. 78 (13), 3484–3496. 10.1158/0008-5472.CAN-17-3454 29588351 PMC8073197

[B116] Marshall K. R. Gong M. Wodke L. Lamb J. H. Jones D. J. Farmer P. B. (2005). The human apoptosis-inducing protein AMID is an oxidoreductase with a modified flavin cofactor and DNA binding activity. J. Biol. Chem. 280 (35), 30735–30740. 10.1074/jbc.M414018200 15958387

[B117] McGill M. R. Lebofsky M. Norris H. R. Slawson M. H. Bajt M. L. Xie Y. (2013). Plasma and liver acetaminophen-protein adduct levels in mice after acetaminophen treatment: dose-response, mechanisms, and clinical implications. Toxicol. Appl. Pharmacol. 269 (3), 240–249. 10.1016/j.taap.2013.03.026 23571099 PMC3654056

[B118] McKie A. T. Barrow D. Latunde-Dada G. O. Rolfs A. Sager G. Mudaly E. (2001). An iron-regulated ferric reductase associated with the absorption of dietary iron. Science 291 (5509), 1755–1759. 10.1126/science.1057206 11230685

[B119] Miotto G. Rossetto M. Di Paolo M. L. Orian L. Venerando R. Roveri A. (2020). Insight into the mechanism of ferroptosis inhibition by ferrostatin-1. Redox Biol. 28, 101328. 10.1016/j.redox.2019.101328 31574461 PMC6812032

[B120] Mishima E. Ito J. Wu Z. Nakamura T. Wahida A. Doll S. (2022). A non-canonical vitamin K cycle is a potent ferroptosis suppressor. Nature 608 (7924), 778–783. 10.1038/s41586-022-05022-3 35922516 PMC9402432

[B121] Mitchell J. R. Jollow D. J. Potter W. Z. Davis D. C. Gillette J. R. Brodie B. B. (1973). Acetaminophen-induced hepatic necrosis. I. Role of drug metabolism. J. Pharmacol. Exp. Ther. 187 (1), 185–194. 4746326

[B122] Moon J. H. Jang H. C. (2022). Gestational diabetes mellitus: diagnostic approaches and maternal-offspring complications. Diabetes Metab. J. 46 (1), 3–14. 10.4093/dmj.2021.0335 35135076 PMC8831816

[B123] Mueller S. Rausch V. (2015). The role of iron in alcohol-mediated hepatocarcinogenesis. Adv. Exp. Med. Biol. 815, 89–112. 10.1007/978-3-319-09614-8_6 25427903

[B124] Muri J. Thut H. Bornkamm G. W. Kopf M. (2019). B1 and marginal zone B cells but not follicular B2 cells require Gpx4 to prevent lipid peroxidation and ferroptosis. Cell Rep. 29 (9), 2731–2744. 10.1016/j.celrep.2019.10.070 31775041

[B125] Nelson J. E. Wilson L. Brunt E. M. Yeh M. M. Kleiner D. E. Unalp-Arida A. (2011). Relationship between the pattern of hepatic iron deposition and histological severity in nonalcoholic fatty liver disease. Hepatology 53 (2), 448–457. 10.1002/hep.24038 21274866 PMC3058264

[B126] Nemeth E. Ganz T. (2006). Regulation of iron metabolism by hepcidin. Annu. Rev. Nutr. 26, 323–342. 10.1146/annurev.nutr.26.061505.111303 16848710

[B127] Ng S. W. Norwitz S. G. Norwitz E. R. (2019). The impact of iron overload and ferroptosis on reproductive disorders in humans: implications for preeclampsia. Int. J. Mol. Sci. 20 (13), 3283. 10.3390/ijms20133283 31277367 PMC6651445

[B128] Nguyen H. P. Yi D. Lin F. Viscarra J. A. Tabuchi C. Ngo K. (2020). Aifm2, a NADH oxidase, supports robust glycolysis and is required for cold- and diet-induced thermogenesis. Mol. Cell 77 (3), 600–617. 10.1016/j.molcel.2019.12.002 31952989 PMC7031813

[B129] Ni H. Qin H. Sun C. Liu Y. Ruan G. Guo Q. (2021). MiR-375 reduces the stemness of gastric cancer cells through triggering ferroptosis. Stem Cell Res. Ther. 12 (1), 325. 10.1186/s13287-021-02394-7 34090492 PMC8180146

[B130] Nickkholgh A. Barro-Bejarano M. Liang R. Zorn M. Mehrabi A. Gebhard M. M. (2008). Signs of reperfusion injury following CO2 pneumoperitoneum: an *in vivo* microscopy study. Surg. Endosc. 22 (1), 122–128. 10.1007/s00464-007-9386-6 17483991

[B131] Niki E. (2009). Lipid peroxidation: physiological levels and dual biological effects. Free Radic. Biol. Med. 47 (5), 469–484. 10.1016/j.freeradbiomed.2009.05.032 19500666

[B132] Niu B. Lei X. Xu Q. Ju Y. Xu D. Mao L. (2022). Protecting mitochondria via inhibiting VDAC1 oligomerization alleviates ferroptosis in acetaminophen-induced acute liver injury. Cell Biol. Toxicol. 38 (3), 505–530. 10.1007/s10565-021-09624-x 34401974

[B133] Ohgami R. S. Campagna D. R. Greer E. L. Antiochos B. McDonald A. Chen J. (2005). Identification of a ferrireductase required for efficient transferrin-dependent iron uptake in erythroid cells. Nat. Genet. 37 (11), 1264–1269. 10.1038/ng1658 16227996 PMC2156108

[B134] Okuda M. Li K. Beard M. R. Showalter L. A. Scholle F. Lemon S. M. (2002). Mitochondrial injury, oxidative stress, and antioxidant gene expression are induced by hepatitis C virus core protein. Gastroenterology 122 (2), 366–375. 10.1053/gast.2002.30983 11832451

[B135] Ooko E. Saeed M. E. Kadioglu O. Sarvi S. Colak M. Elmasaoudi K. (2015). Artemisinin derivatives induce iron-dependent cell death (ferroptosis) in tumor cells. Phytomedicine 22 (11), 1045–1054. 10.1016/j.phymed.2015.08.002 26407947

[B136] Orozco-Aguilar J. Simon F. Cabello-Verrugio C. (2021). Redox-dependent effects in the physiopathological role of bile acids. Oxid. Med. Cell Longev. 2021, 4847941. 10.1155/2021/4847941 34527174 PMC8437588

[B137] Outten F. W. Theil E. C. (2009). Iron-based redox switches in biology. Antioxid. Redox Signal 11 (5), 1029–1046. 10.1089/ars.2008.2296 19021503 PMC2842161

[B138] Pavličev M. Wagner G. P. Chavan A. R. Owens K. Maziarz J. Dunn-Fletcher C. (2017). Single-cell transcriptomics of the human placenta: inferring the cell communication network of the maternal-fetal interface. Genome Res. 27 (3), 349–361. 10.1101/gr.207597.116 28174237 PMC5340963

[B139] Perez M. J. Macias R. I. Marin J. J. (2006). Maternal cholestasis induces placental oxidative stress and apoptosis. Protective effect of ursodeoxycholic acid. Placenta 27 (1), 34–41. 10.1016/j.placenta.2004.10.020 16310035

[B140] Plays M. Müller S. Rodriguez R. (2021). Chemistry and biology of ferritin. Metallomics. 13 (5), mfab021. 10.1093/mtomcs/mfab021 33881539 PMC8083198

[B141] Protchenko O. Baratz E. Jadhav S. Li F. Shakoury-Elizeh M. Gavrilova O. (2021). Iron chaperone poly rC binding protein 1 protects mouse liver from lipid peroxidation and steatosis. Hepatology 73 (3), 1176–1193. 10.1002/hep.31328 32438524 PMC8364740

[B142] Qi J. Kim J. W. Zhou Z. Lim C. W. Kim B. (2020). Ferroptosis affects the progression of nonalcoholic steatohepatitis via the modulation of lipid peroxidation-mediated cell death in mice. Am. J. Pathol. 190 (1), 68–81. 10.1016/j.ajpath.2019.09.011 31610178

[B143] Qi W. Li Z. Xia L. Dai J. Zhang Q. Wu C. (2019). LncRNA GABPB1-AS1 and GABPB1 regulate oxidative stress during erastin-induced ferroptosis in HepG2 hepatocellular carcinoma cells. Sci. Rep. 9 (1), 16185. 10.1038/s41598-019-52837-8 31700067 PMC6838315

[B144] Raper N. R. Rosenthal J. C. Woteki C. E. (1984). Estimates of available iron in diets of individuals 1 year old and older in the Nationwide Food Consumption Survey. J. Am. Diet. Assoc. 84 (7), 783–787. 10.1016/s0002-8223(21)08245-6 6736505

[B145] Rayman M. P. Barlis J. Evans R. W. Redman C. W. King L. J. (2002). Abnormal iron parameters in the pregnancy syndrome preeclampsia. Am. J. Obstet. Gynecol. 187 (2), 412–418. 10.1067/mob.2002.123895 12193935

[B146] Reyes H. Báez M. E. González M. C. Hernández I. Palma J. Ribalta J. (2000). Selenium, zinc and copper plasma levels in intrahepatic cholestasis of pregnancy, in normal pregnancies and in healthy individuals, in Chile. J. Hepatol. 32 (4), 542–549. 10.1016/s0168-8278(00)80214-7 10782901

[B147] Rinella M. E. Neuschwander-Tetri B. A. Siddiqui M. S. Abdelmalek M. F. Caldwell S. Barb D. (2023). AASLD Practice Guidance on the clinical assessment and management of nonalcoholic fatty liver disease. Hepatology 77 (5), 1797–1835. 10.1097/HEP.0000000000000323 36727674 PMC10735173

[B148] Roh J. L. Kim E. H. Jang H. Shin D. (2017). Nrf2 inhibition reverses the resistance of cisplatin-resistant head and neck cancer cells to artesunate-induced ferroptosis. Redox Biol. 11, 254–262. 10.1016/j.redox.2016.12.010 28012440 PMC5198738

[B149] Rouzer C. A. Marnett L. J. (2003). Mechanism of free radical oxygenation of polyunsaturated fatty acids by cyclooxygenases. Chem. Rev. 103 (6), 2239–2304. 10.1021/cr000068x 12797830

[B150] Rui L. (2014). Energy metabolism in the liver. Compr. Physiol. 4 (1), 177–197. 10.1002/cphy.c130024 24692138 PMC4050641

[B151] Rushing G. D. Britt L. D. (2008). Reperfusion injury after hemorrhage: a collective review. Ann. Surg. 247 (6), 929–937. 10.1097/SLA.0b013e31816757f7 18520219

[B152] Sangkhae V. Fisher A. L. Chua K. J. Ruchala P. Ganz T. Nemeth E. (2020). Maternal hepcidin determines embryo iron homeostasis in mice. Blood 136 (19), 2206–2216. 10.1182/blood.2020005745 32584957 PMC7645983

[B153] Sangkhae V. Nemeth E. (2019). Placental iron transport: the mechanism and regulatory circuits. Free Radic. Biol. Med. 133, 254–261. 10.1016/j.freeradbiomed.2018.07.001 29981833 PMC7059975

[B154] Schoots M. H. Gordijn S. J. Scherjon S. A. van Goor H. Hillebrands J. L. (2018). Oxidative stress in placental pathology. Placenta 69, 153–161. 10.1016/j.placenta.2018.03.003 29622278

[B155] Schwabe R. F. Luedde T. (2018). Apoptosis and necroptosis in the liver: a matter of life and death. Nat. Rev. Gastroenterol. Hepatol. 15 (12), 738–752. 10.1038/s41575-018-0065-y 30250076 PMC6490680

[B156] Seiler A. Schneider M. Förster H. Roth S. Wirth E. K. Culmsee C. (2008). Glutathione peroxidase 4 senses and translates oxidative stress into 12/15-lipoxygenase dependent- and AIF-mediated cell death. Cell Metab. 8 (3), 237–248. 10.1016/j.cmet.2008.07.005 18762024

[B157] Shah R. Shchepinov M. S. Pratt D. A. (2018). Resolving the role of lipoxygenases in the initiation and execution of ferroptosis. ACS Cent. Sci. 4 (3), 387–396. 10.1021/acscentsci.7b00589 29632885 PMC5879472

[B158] Shaw J. Chakraborty A. Nag A. Chattopadyay A. Dasgupta A. K. Bhattacharyya M. (2017). Intracellular iron overload leading to DNA damage of lymphocytes and immune dysfunction in thalassemia major patients. Eur. J. Haematol. 99 (5), 399–408. 10.1111/ejh.12936 28815805

[B159] Shen Z. Song J. Yung B. C. Zhou Z. Wu A. Chen X. (2018). Emerging strategies of cancer therapy based on ferroptosis. Adv. Mat. 30 (12), e1704007. 10.1002/adma.201704007 PMC6377162 29356212

[B160] Shimada K. Skouta R. Kaplan A. Yang W. S. Hayano M. Dixon S. J. (2016). Global survey of cell death mechanisms reveals metabolic regulation of ferroptosis. Nat. Chem. Biol. 12 (7), 497–503. 10.1038/nchembio.2079 27159577 PMC4920070

[B161] Siegel R. L. Miller K. D. Wagle N. S. Jemal A. (2023). Cancer statistics, 2023. CA Cancer J. Clin. 73 (1), 17–48. 10.3322/caac.21763 36633525

[B162] Sokol R. J. McKim J. M. Jr. Goff M. C. Ruyle S. Z. Devereaux M. W. Han D. (1998). Vitamin E reduces oxidant injury to mitochondria and the hepatotoxicity of taurochenodeoxycholic acid in the rat. Gastroenterology 114 (1), 164–174. 10.1016/s0016-5085(98)70644-4 9428230

[B163] Sokol R. J. Straka M. S. Dahl R. Devereaux M. W. Yerushalmi B. Gumpricht E. (2001). Role of oxidant stress in the permeability transition induced in rat hepatic mitochondria by hydrophobic bile acids. Pediatr. Res. 49 (4), 519–531. 10.1203/00006450-200104000-00014 11264436

[B164] Song Z. Jia G. Ma P. Cang S. (2021). Exosomal miR-4443 promotes cisplatin resistance in non-small cell lung carcinoma by regulating FSP1 m6A modification-mediated ferroptosis. Life Sci. 276, 119399. 10.1016/j.lfs.2021.119399 33781830

[B165] Soula M. Weber R. A. Zilka O. Alwaseem H. La K. Yen F. (2020). Metabolic determinants of cancer cell sensitivity to canonical ferroptosis inducers. Nat. Chem. Biol. 16 (12), 1351–1360. 10.1038/s41589-020-0613-y 32778843 PMC8299533

[B166] Stockwell B. R. Jiang X. (2019). A physiological function for ferroptosis in tumor suppression by the immune system. Cell Metab. 30 (1), 14–15. 10.1016/j.cmet.2019.06.012 31269423 PMC6944065

[B167] Stravitz R. T. Lee W. M. (2019). Acute liver failure. Lancet 394 (10201), 869–881. 10.1016/S0140-6736(19)31894-X 31498101 PMC10836844

[B168] Su H. Liu Y. Huang J. (2023). Ferroptosis-related gene SLC1A5 is a novel prognostic biomarker and correlates with immune microenvironment in HBV-related HCC. J. Clin. Med. 12 (5), 1715. 10.3390/jcm12051715 36902506 PMC10003624

[B169] Sun C. Wu Q. J. Gao S. Y. Ma Z. M. Liu Y. S. Zhang J. Y. (2020). Association between the ferritin level and risk of gestational diabetes mellitus: a meta-analysis of observational studies. J. Diabetes Investig. 11 (3), 707–718. 10.1111/jdi.13170 PMC7232272 31667982

[B170] Sun X. Niu X. Chen R. He W. Chen D. Kang R. (2016). Metallothionein-1G facilitates sorafenib resistance through inhibition of ferroptosis. Hepatology 64 (2), 488–500. 10.1002/hep.28574 27015352 PMC4956496

[B171] Sun X. Ou Z. Xie M. Kang R. Fan Y. Niu X. (2015). HSPB1 as a novel regulator of ferroptotic cancer cell death. Oncogene 34 (45), 5617–5625. 10.1038/onc.2015.32 25728673 PMC4640181

[B172] Sunny N. E. Parks E. J. Browning J. D. Burgess S. C. (2011). Excessive hepatic mitochondrial TCA cycle and gluconeogenesis in humans with nonalcoholic fatty liver disease. Cell Metab. 14 (6), 804–810. 10.1016/j.cmet.2011.11.004 22152305 PMC3658280

[B173] Takimoto E. Champion H. C. Li M. Ren S. Rodriguez E. R. Tavazzi B. (2005). Oxidant stress from nitric oxide synthase-3 uncoupling stimulates cardiac pathologic remodeling from chronic pressure load. J. Clin. Invest. 115 (5), 1221–1231. 10.1172/JCI21968 15841206 PMC1077169

[B174] Tang D. Chen X. Kang R. Kroemer G. (2021). Ferroptosis: molecular mechanisms and health implications. Cell Res. 31 (2), 107–125. 10.1038/s41422-020-00441-1 33268902 PMC8026611

[B175] Tao W. Wang N. Ruan J. Cheng X. Fan L. Zhang P. (2022). Enhanced ROS-boosted phototherapy against pancreatic cancer via nrf2-mediated stress-defense pathway suppression and ferroptosis induction. ACS Appl. Mater Interfaces 14 (5), 6404–6416. 10.1021/acsami.1c22861 35077153

[B176] Tegeder I. Costigan M. Griffin R. S. Abele A. Belfer I. Schmidt H. (2006). GTP cyclohydrolase and tetrahydrobiopterin regulate pain sensitivity and persistence. Nat. Med. 12 (11), 1269–1277. 10.1038/nm1490 17057711

[B177] Tesfay L. Paul B. T. Konstorum A. Deng Z. Cox A. O. Lee J. (2019). Stearoyl-CoA desaturase 1 protects ovarian cancer cells from ferroptotic cell death. Cancer Res. 79 (20), 5355–5366. 10.1158/0008-5472.CAN-19-0369 31270077 PMC6801059

[B178] Tian P. Xu Z. Guo J. Zhao J. Chen W. Huang W. (2024). Hypoxia causes trophoblast cell ferroptosis to induce miscarriage through lnc-HZ06/HIF1α-SUMO/NCOA4 axis. Redox Biol. 70, 103073. 10.1016/j.redox.2024.103073 38335622 PMC10869313

[B179] Tirmenstein M. A. Nelson S. D. (1990). Acetaminophen-induced oxidation of protein thiols. Contribution of impaired thiol-metabolizing enzymes and the breakdown of adenine nucleotides. J. Biol. Chem. 265 (6), 3059–3065. 10.1016/s0021-9258(19)39733-9 2303440

[B180] Tokushige K. Ikejima K. Ono M. Eguchi Y. Kamada Y. Itoh Y. (2021). Evidence-based clinical practice guidelines for nonalcoholic fatty liver disease/nonalcoholic steatohepatitis 2020. J. Gastroenterol. 56 (11), 951–963. 10.1007/s00535-021-01796-x 34533632 PMC8531062

[B181] Torti S. V. Torti F. M. (2013). Iron and cancer: more ore to be mined. Nat. Rev. Cancer 13 (5), 342–355. 10.1038/nrc3495 23594855 PMC4036554

[B182] Tripathi A. K. Haldar S. Qian J. Beserra A. Suda S. Singh A. (2015). Prion protein functions as a ferrireductase partner for ZIP14 and DMT1. Free Radic. Biol. Med. 84, 322–330. 10.1016/j.freeradbiomed.2015.03.037 25862412 PMC4476631

[B183] Tschuck J. Theilacker L. Rothenaigner I. Weiß S. A. I. Akdogan B. Lam V. T. (2023). Farnesoid X receptor activation by bile acids suppresses lipid peroxidation and ferroptosis. Nat. Commun. 14 (1), 6908. 10.1038/s41467-023-42702-8 37903763 PMC10616197

[B184] Tsurusaki S. Tsuchiya Y. Koumura T. Nakasone M. Sakamoto T. Matsuoka M. (2019). Hepatic ferroptosis plays an important role as the trigger for initiating inflammation in nonalcoholic steatohepatitis. Cell Death Dis. 10 (6), 449. 10.1038/s41419-019-1678-y 31209199 PMC6579767

[B185] Ufer C. Borchert A. Kuhn H. (2003). Functional characterization of cis- and trans-regulatory elements involved in expression of phospholipid hydroperoxide glutathione peroxidase. Nucleic Acids Res. 31 (15), 4293–4303. 10.1093/nar/gkg650 12888488 PMC169948

[B186] Valenti L. Dongiovanni P. Fargion S. (2012). Diagnostic and therapeutic implications of the association between ferritin level and severity of nonalcoholic fatty liver disease. World J. Gastroenterol. 18 (29), 3782–3786. 10.3748/wjg.v18.i29.3782 22876027 PMC3413047

[B187] van Santen S. Kroot J. J. Zijderveld G. Wiegerinck E. T. Spaanderman M. E. Swinkels D. W. (2013). The iron regulatory hormone hepcidin is decreased in pregnancy: a prospective longitudinal study. Clin. Chem. Lab. Med. 51 (7), 1395–1401. 10.1515/cclm-2012-0576 23241678

[B188] Venkatesh D. O'Brien N. A. Zandkarimi F. Tong D. R. Stokes M. E. Dunn D. E. (2020). MDM2 and MDMX promote ferroptosis by PPARα-mediated lipid remodeling. Genes Dev. 34 (7-8), 526–543. 10.1101/gad.334219.119 32079652 PMC7111265

[B189] Vounzoulaki E. Khunti K. Abner S. C. Tan B. K. Davies M. J. Gillies C. L. (2020). Progression to type 2 diabetes in women with a known history of gestational diabetes: systematic review and meta-analysis. Bmj 369, m1361. 10.1136/bmj.m1361 32404325 PMC7218708

[B190] Wang D. Xie N. Gao W. Kang R. Tang D. (2018). The ferroptosis inducer erastin promotes proliferation and differentiation in human peripheral blood mononuclear cells. Biochem. Biophys. Res. Commun. 503 (3), 1689–1695. 10.1016/j.bbrc.2018.07.100 30049441 PMC6179365

[B191] Wang L. Zhang Z. Li M. Wang F. Jia Y. Zhang F. (2019b). P53-dependent induction of ferroptosis is required for artemether to alleviate carbon tetrachloride-induced liver fibrosis and hepatic stellate cell activation. IUBMB Life 71 (1), 45–56. 10.1002/iub.1895 30321484

[B192] Wang W. Green M. Choi J. E. Gijón M. Kennedy P. D. Johnson J. K. (2019a). CD8(+) T cells regulate tumour ferroptosis during cancer immunotherapy. Nature 569 (7755), 270–274. 10.1038/s41586-019-1170-y 31043744 PMC6533917

[B193] Wang Y. Zhao M. Zhao L. Geng Y. Li G. Chen L. (2023). HBx-induced HSPA8 stimulates HBV replication and suppresses ferroptosis to support liver cancer progression. Cancer Res. 83 (7), 1048–1061. 10.1158/0008-5472.CAN-22-3169 36745032

[B194] Wei W. Hu Y. Y. (2014). Expression of hypoxia-regulated genes and glycometabolic genes in placenta from patients with intrahepatic cholestasis of pregnancy. Placenta 35 (9), 732–736. 10.1016/j.placenta.2014.06.372 25063250

[B195] Wenzel S. E. Tyurina Y. Y. Zhao J. St Croix C. M. Dar H. H. Mao G. (2017). PEBP1 wardens ferroptosis by enabling lipoxygenase generation of lipid death signals. Cell 171 (3), 628–641. 10.1016/j.cell.2017.09.044 29053969 PMC5683852

[B196] Wikström Shemer E. Marschall H. U. Ludvigsson J. F. Stephansson O. (2013). Intrahepatic cholestasis of pregnancy and associated adverse pregnancy and fetal outcomes: a 12-year population-based cohort study. Bjog 120 (6), 717–723. 10.1111/1471-0528.12174 23418899

[B197] Woolbright B. L. Jaeschke H. (2019). Inflammation and cell death during cholestasis: the evolving role of bile acids. Gene Expr. 19 (3), 215–228. 10.3727/105221619X15614873062730 31253204 PMC6827039

[B198] Wu H. Liu A. (2021). Long non-coding RNA NEAT1 regulates ferroptosis sensitivity in non-small-cell lung cancer. J. Int. Med. Res. 49 (3), 300060521996183. 10.1177/0300060521996183 33730930 PMC8166394

[B199] Wu W. B. Xu Y. Y. Cheng W. W. Wang Y. X. Liu Y. Huang D. (2015). Agonist of farnesoid X receptor protects against bile acid induced damage and oxidative stress in mouse placenta--a study on maternal cholestasis model. Placenta 36 (5), 545–551. 10.1016/j.placenta.2015.02.005 25747729

[B200] Wu Z. Geng Y. Lu X. Shi Y. Wu G. Zhang M. (2019). Chaperone-mediated autophagy is involved in the execution of ferroptosis. Proc. Natl. Acad. Sci. U. S. A. 116 (8), 2996–3005. 10.1073/pnas.1819728116 30718432 PMC6386716

[B201] Xu M. Guo D. Gu H. Zhang L. Lv S. (2016). Selenium and preeclampsia: a systematic review and meta-analysis. Biol. Trace Elem. Res. 171 (2), 283–292. 10.1007/s12011-015-0545-7 26516080

[B202] Yadav P. Sharma P. Sundaram S. Venkatraman G. Bera A. K. Karunagaran D. (2021). SLC7A11/xCT is a target of miR-5096 and its restoration partially rescues miR-5096-mediated ferroptosis and anti-tumor effects in human breast cancer cells. Cancer Lett. 522, 211–224. 10.1016/j.canlet.2021.09.033 34571083

[B203] Yagoda N. von Rechenberg M. Zaganjor E. Bauer A. J. Yang W. S. Fridman D. J. (2007). RAS-RAF-MEK-dependent oxidative cell death involving voltage-dependent anion channels. Nature 447 (7146), 864–868. 10.1038/nature05859 17568748 PMC3047570

[B204] Yamada N. Karasawa T. Wakiya T. Sadatomo A. Ito H. Kamata R. (2020). Iron overload as a risk factor for hepatic ischemia-reperfusion injury in liver transplantation: potential role of ferroptosis. Am. J. Transpl. 20 (6), 1606–1618. 10.1111/ajt.15773 31909544

[B205] Yamane D. Hayashi Y. Matsumoto M. Nakanishi H. Imagawa H. Kohara M. (2022). FADS2-dependent fatty acid desaturation dictates cellular sensitivity to ferroptosis and permissiveness for hepatitis C virus replication. Cell Chem. Biol. 29 (5), 799–810.e4. 10.1016/j.chembiol.2021.07.022 34520742 PMC8913804

[B206] Yan B. Ai Y. Sun Q. Ma Y. Cao Y. Wang J. (2021). Membrane damage during ferroptosis is caused by oxidation of phospholipids catalyzed by the oxidoreductases POR and CYB5R1. Mol. Cell 81 (2), 355–369.e10. 10.1016/j.molcel.2020.11.024 33321093

[B207] Yang K. Yang Y. Pan B. Fu S. Cheng J. Liu J. (2022a). Relationship between iron metabolism and gestational diabetes mellitus: a systemic review and meta analysis. Asia Pac J. Clin. Nutr. 31 (2), 242–254. 10.6133/apjcn.202206_31(2).0010 35766560

[B208] Yang Q. Jian J. Katz S. Abramson S. B. Huang X. (2012). 17β-Estradiol inhibits iron hormone hepcidin through an estrogen responsive element half-site. Endocrinology 153 (7), 3170–3178. 10.1210/en.2011-2045 22535765 PMC3380311

[B209] Yang W. S. SriRamaratnam R. Welsch M. E. Shimada K. Skouta R. Viswanathan V. S. (2014). Regulation of ferroptotic cancer cell death by GPX4. Cell. 156 (1-2), 317–331. 10.1016/j.cell.2013.12.010 24439385 PMC4076414

[B210] Yang W. S. Stockwell B. R. (2008). Synthetic lethal screening identifies compounds activating iron-dependent, nonapoptotic cell death in oncogenic-RAS-harboring cancer cells. Chem. Biol. 15 (3), 234–245. 10.1016/j.chembiol.2008.02.010 18355723 PMC2683762

[B211] Yang Y. Wang Y. Guo L. Gao W. Tang T. L. Yan M. (2022b). Interaction between macrophages and ferroptosis. Cell Death Dis. 13 (4), 355. 10.1038/s41419-022-04775-z 35429990 PMC9013379

[B212] Yao Y. Chen Z. Zhang H. Chen C. Zeng M. Yunis J. (2021). Selenium-GPX4 axis protects follicular helper T cells from ferroptosis. Nat. Immunol. 22 (9), 1127–1139. 10.1038/s41590-021-00996-0 34413521

[B213] Yeh K. Y. Yeh M. Glass J. (2011). Interactions between ferroportin and hephaestin in rat enterocytes are reduced after iron ingestion. Gastroenterology 141 (1), 292–299. 10.1053/j.gastro.2011.03.059 21473866

[B214] Yin H. Xu L. Porter N. A. (2011). Free radical lipid peroxidation: mechanisms and analysis. Chem. Rev. 111 (10), 5944–5972. 10.1021/cr200084z 21861450

[B215] You M. Jogasuria A. Taylor C. Wu J. (2015). Sirtuin 1 signaling and alcoholic fatty liver disease. Hepatobiliary Surg. Nutr. 4 (2), 88–100. 10.3978/j.issn.2304-3881.2014.12.06 26005675 PMC4405418

[B216] Young M. F. Griffin I. Pressman E. McIntyre A. W. Cooper E. McNanley T. (2012). Maternal hepcidin is associated with placental transfer of iron derived from dietary heme and nonheme sources. J. Nutr. 142 (1), 33–39. 10.3945/jn.111.145961 22113871 PMC3237230

[B217] Younossi Z. Anstee Q. M. Marietti M. Hardy T. Henry L. Eslam M. (2018). Global burden of NAFLD and NASH: trends, predictions, risk factors and prevention. Nat. Rev. Gastroenterol. Hepatol. 15 (1), 11–20. 10.1038/nrgastro.2017.109 28930295

[B218] Yu Y. Jiang L. Wang H. Shen Z. Cheng Q. Zhang P. (2020). Hepatic transferrin plays a role in systemic iron homeostasis and liver ferroptosis. Blood 136 (6), 726–739. 10.1182/blood.2019002907 32374849 PMC7414596

[B219] Yuan L. Kaplowitz N. (2013). Mechanisms of drug-induced liver injury. Clin. Liver Dis. 17 (4), 507–518. vii. 10.1016/j.cld.2013.07.002 24099014 PMC3793205

[B220] Zeng T. Deng G. Zhong W. Gao Z. Ma S. Mo C. (2020). Indoleamine 2, 3-dioxygenase 1enhanceshepatocytes ferroptosis in acute immune hepatitis associated with excess nitrative stress. Free Radic. Biol. Med. 152, 668–679. 10.1016/j.freeradbiomed.2020.01.009 31945497

[B221] Zhang H. He Y. Wang J. X. Chen M. H. Xu J. J. Jiang M. H. (2020). miR-30-5p-mediated ferroptosis of trophoblasts is implicated in the pathogenesis of preeclampsia. Redox Biol. 29, 101402. 10.1016/j.redox.2019.101402 31926626 PMC6928320

[B222] Zhang Q. Fan X. Zhang X. Ju S. (2023). Ferroptosis in tumors and its relationship to other programmed cell death: role of non-coding RNAs. J. Transl. Med. 21 (1), 514. 10.1186/s12967-023-04370-6 37516888 PMC10387214

[B223] Zhao Y. Gao Q. Li B. Wang Y. Wang Y. (2023). Ferroptosis and its potential role in gestational diabetes mellitus: updated evidence from pathogenesis to therapy. Front. Endocrinol. (Lausanne) 14, 1177547. 10.3389/fendo.2023.1177547 37664858 PMC10471987

[B224] Zheng J. Conrad M. (2020). The metabolic underpinnings of ferroptosis. Cell Metab. 32 (6), 920–937. 10.1016/j.cmet.2020.10.011 33217331

[B225] Zhou Z. Ye T. J. Bonavita G. Daniels M. Kainrad N. Jogasuria A. (2019). Adipose-specific lipin-1 overexpression renders hepatic ferroptosis and exacerbates alcoholic steatohepatitis in mice. Hepatol. Commun. 3 (5), 656–669. 10.1002/hep4.1333 31061954 PMC6492478

[B226] Zhou Z. Ye T. J. DeCaro E. Buehler B. Stahl Z. Bonavita G. (2020). Intestinal SIRT1 deficiency protects mice from ethanol-induced liver injury by mitigating ferroptosis. Am. J. Pathol. 190 (1), 82–92. 10.1016/j.ajpath.2019.09.012 31610175 PMC6943377

[B227] Zhu L. Chen D. Zhu Y. Pan T. Xia D. Cai T. (2021). GPX4-Regulated ferroptosis mediates S100-induced experimental autoimmune hepatitis associated with the Nrf2/HO-1 signaling pathway. Oxid. Med. Cell Longev. 2021, 6551069. 10.1155/2021/6551069 34966478 PMC8712167

[B228] Zhu P. Zhao S. M. Li Y. Z. Guo H. Wang L. Tian P. (2019). Correlation of lipid peroxidation and ATP enzyme on erythrocyte membrane with fetal distress in the uterus in patients with intrahepatic cholestasis of pregnancy. Eur. Rev. Med. Pharmacol. Sci. 23 (6), 2318–2324. 10.26355/eurrev_201903_17371 30964154

[B229] Zou Y. Li H. Graham E. T. Deik A. A. Eaton J. K. Wang W. (2020). Cytochrome P450 oxidoreductase contributes to phospholipid peroxidation in ferroptosis. Nat. Chem. Biol. 16 (3), 302–309. 10.1038/s41589-020-0472-6 32080622 PMC7353921

[B230] Zuo Y. B. Zhang Y. F. Zhang R. Tian J. W. Lv X. B. Li R. (2022). Ferroptosis in cancer progression: role of noncoding RNAs. Int. J. Biol. Sci. 18 (5), 1829–1843. 10.7150/ijbs.66917 35342359 PMC8935228

